# Multicenter Privacy‐Preserving Federated Models for Predicting Postoperative Delirium and Acute Kidney Injury in Older Patients

**DOI:** 10.1002/mco2.70944

**Published:** 2026-08-22

**Authors:** Qian Wang, Yu‐xiang Song, Xiao‐dong Yang, Jing‐wei Zhang, Rui‐zhe Sun, Xiao‐dong Wu, Ao Li, Jing‐sheng Lou, Hao Li, Yan‐hong Liu, Jun‐mei Xu, Di‐fen Wang, Qing‐ping Wu, Yu‐ming Peng, Yi‐qiang Chen, Jiang‐bei Cao, Wei‐dong Mi

**Affiliations:** ^1^ Department of Anaesthesiology The First Medical Center of Chinese PLA General Hospital Beijing China; ^2^ Chinese PLA Medical School Beijing China; ^3^ Beijing Key Laboratory of Perioperative Multimodal Digital Intelligence‐Integrated Diagnosis and Treatment Beijing China; ^4^ National Clinical Research Center for Geriatric Diseases Chinese PLA General Hospital Beijing China; ^5^ Institute of Computing Technology, Chinese Academy of Sciences Beijing China; ^6^ Medical School, Tianjin University Tianjin China; ^7^ Department of Anaesthesiology The Second Xiangya Hospital of Central South University Changsha China; ^8^ Department of Intensive Care Medicine The Affiliated Hospital of Guizhou Medical University Guiyang China; ^9^ Department of Anaesthesiology Union Hospital, Tongji Medical College, Huazhong University of Science and Technology Wuhan China; ^10^ Department of Anaesthesiology Beijing Tiantan Hospital Capital Medical University Beijing China

**Keywords:** acute kidney injury, federated learning, multicenter study, older adults, postoperative complications, postoperative delirium

## Abstract

Postoperative delirium (POD) and acute kidney injury (AKI) are serious complications in older patients undergoing surgery, yet predictive model development is often constrained by single‐center data limitations and privacy concerns that preclude centralized data sharing. To address these challenges, we retrospectively evaluated a simulated federated learning (FL) framework using multicenter datasets partitioned by hospital source, without sharing raw patient data across centers. A total of 7,216 non‐cardiac, non‐neurosurgical patients aged 65 years or older were included across five centers, with four contributing training data and one serving as an external validation site. Using a multilayer perceptron architecture, we implemented three federated algorithms and benchmarked them against local learning models (LLMs) and centralized learning models (CLMs). For POD, federated learning models (FLMs) achieved internal area under the curve (AUC) values of 0.725‐0.726 and external AUCs of 0.700‐0.701. For AKI, internal AUCs reached 0.780 and external AUCs ranged from 0.740 to 0.741. FLM performance was statistically comparable to CLMs (*p* > 0.05). These findings support federated learning as a feasible privacy‐preserving strategy that achieved discrimination comparable to centralized learning for multicenter prediction of postoperative complications in older patients.

## Graphical abstract

Using data from five medical centers, this study retrospectively evaluated a simulated federated learning framework for predicting postoperative delirium and acute kidney injury in older surgical patients. Federated learning achieved performance comparable to centralized learning without direct exchange of raw patient data.

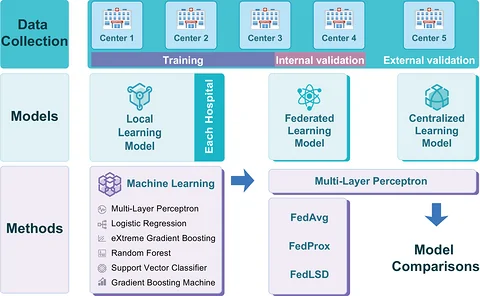

## 1 Introduction

Older adults undergoing surgery are at increased risk of postoperative complications because of diminished physiological reserve, multimorbidity, frailty, and heightened vulnerability to perioperative stress. Among these complications, postoperative delirium (POD) and acute kidney injury (AKI) are two of the most frequent and clinically important adverse events [1, 2]. POD is associated with prolonged hospitalization, functional decline, loss of independence, cognitive deterioration, and increased mortality [3, 4, 5], whereas AKI is linked to longer hospital stay, greater resource utilization, progression to chronic kidney disease, and increased short‐ and long‐term mortality [6, 7]. Although POD and AKI differ in pathophysiology, clinical presentation, and perioperative management, both are highly relevant in older surgical patients and may be mitigated through timely identification of high‐risk individuals. Thus, accurate preoperative or early perioperative risk prediction for each complication is essential to support individualized prevention, monitoring, and resource allocation.

Over the past decade, numerous prediction models for POD or AKI have been developed using conventional statistical methods and, more recently, machine learning (ML) techniques [8, 9, 10]. These models typically integrate demographic characteristics, comorbidities, laboratory indices, medication exposure, and intraoperative variables to estimate perioperative risk [11, 12]. However, their clinical applicability remains limited. Many models were derived from single‐center cohorts or relatively homogeneous populations and lacked rigorous external validation [13, 14, 15]. Consequently, apparent strong performance in development datasets may not translate well across institutions with different patient case mixes, perioperative workflows, surgical profiles, and outcome ascertainment practices [16].

Developing robust multicenter prediction models for perioperative complications is further complicated by growing barriers to centralized data sharing. Regulatory requirements, institutional governance policies, privacy concerns, and heterogeneity in electronic health record systems often restrict direct pooling of patient‐level data across hospitals [17, 18, 19]. In perioperative research, these challenges are amplified by the need to integrate information spanning preoperative assessment, intraoperative management, laboratory testing, and postoperative surveillance, all of which may differ substantially across centers [20]. In addition, multicenter clinical data are often non‐independent and non‐identically distributed (non‐IID), reflecting differences in patient selection, surgical complexity, anesthetic practice, monitoring intensity, and documentation patterns [21, 22, 23]. Under such conditions, traditional centralized ML pipelines may be difficult to implement and may remain vulnerable to site‐specific bias, thereby limiting model transportability and real‐world utility [24].

Federated learning (FL) offers a decentralized paradigm for collaborative model development that allows participating centers to train a shared model without transferring raw patient data to a central repository [25]. By preserving data within institutional boundaries, FL can facilitate multicenter collaboration while reducing privacy, governance, and re‐identification risks [26, 27]. Prior studies have demonstrated the feasibility of FL in several medical domains, including neuroimaging, oncology, dermatology, and other risk stratification tasks [28, 29, 30, 31]. Compared with isolated local learning models (LLMs), FL may improve generalizability by leveraging broader institutional diversity, while compared with centralized learning models (CLMs), it offers a privacy‐preserving alternative that may be more feasible in real‐world healthcare settings. However, important challenges remain, including inter‐site heterogeneity, imbalanced sample sizes, and variation in local feature distributions and outcome prevalence. In the perioperative setting, multicenter FL applications remain scarce, and evidence is particularly limited for separate prediction of POD and AKI in older surgical populations.

To address these gaps, we conducted a multicenter study across five hospitals and retrospectively evaluated a simulated federated learning framework for patients aged ≥ 65 years undergoing non‐cardiac, non‐neurosurgical procedures. We developed and validated separate federated learning models (FLMs) for POD and AKI, and implemented three federated strategies: federated averaging (FedAvg), federated proximal optimization (FedProx), and federated local self‐distillation (FedLSD). We benchmarked these federated models against site‐specific LLMs and CLMs, and evaluated their discrimination, calibration, and clinical utility using decision curve analysis (DCA). In addition, we applied Shapley Additive Explanations (SHAP) to identify influential perioperative predictors and to explore both shared and center‐specific feature importance patterns across institutions and across the two outcomes. By separately modeling POD and AKI within a privacy‐preserving multicenter framework, this study aims to provide a scalable and interpretable approach for perioperative risk stratification in older adults and to support more targeted prevention and management strategies for these two major complications.

## 2 Results

### 2.1 Baseline Characteristics of the Patients

**FIGURE 1:**
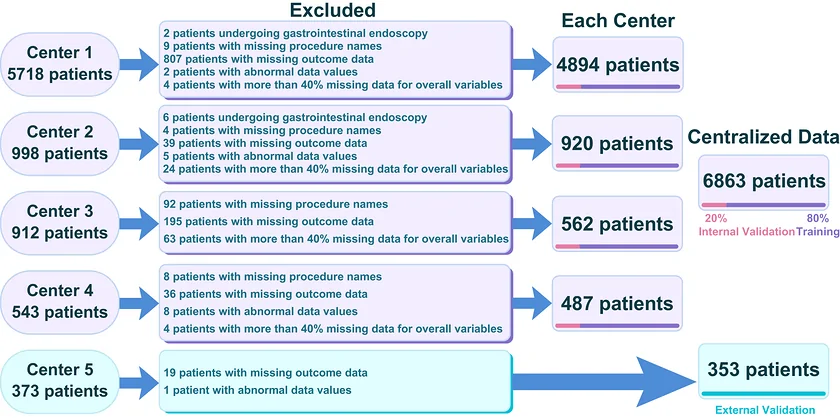
Flowchart of dataset development for training, internal validation, and external validation.

The development cohort comprised four centers, with 5,718, 998, 912, and 543 patients initially enrolled, respectively. After applying exclusion criteria, 4,894, 920, 562, and 487 patients remained for analysis. The external validation center initially enrolled 373 patients, with 353 remaining after exclusions (Figure 1). Before imputation, substantial between‐center differences were observed in the distribution of key demographic, clinical, perioperative, and outcome variables across the five participating centers (Table S1). When pooling the data together, the training set consisted of 5,489 patients, and the internal validation set included 1,374 patients. The incidence of POD was 13.8% (756/5,489) in the training set, 13.8% (189/1,374) in the internal validation set, and 5.7% (20/353) in the external validation set (Table S2). Corresponding incidence rates of AKI were 5.3% (292/5,489), 5.3% (73/1,374), and 6.2% (22/353), respectively (Table S3).

### 2.2 POD Local Learning Models Based on Different ML Algorithms

**FIGURE 2:**
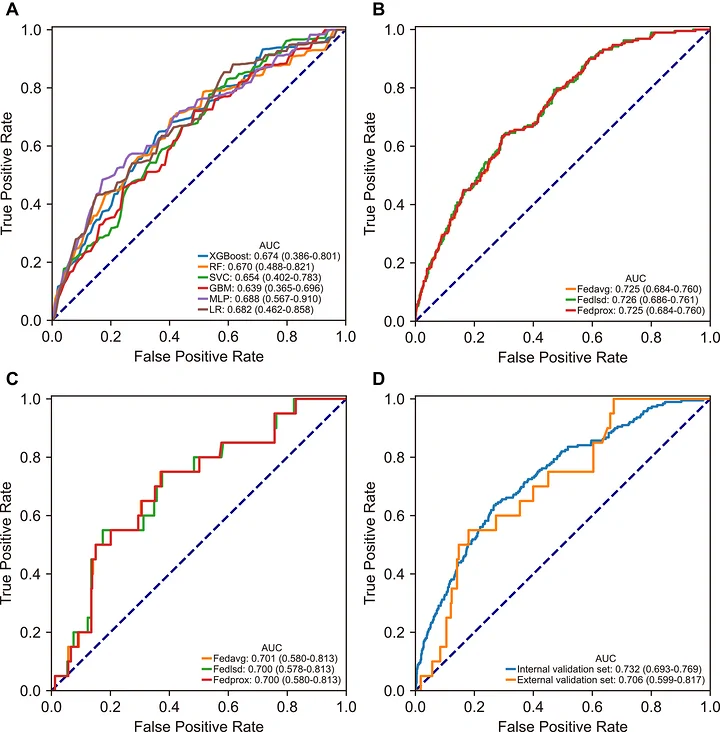
(A) Average ROC curves of POD LLMs based on different machine learning methods across four centers. (B) Internal validation ROC curves of POD FLMs. (C) External validation ROC curves of POD FLMs. (D) ROC curves of POD CLMs.

The internal validation results of LLMs for predicting POD were evaluated across four centers using six different ML algorithms. Figure 2A presents the receiver operating characteristic (ROC) curves aggregated across the four centers together with the averaged area under the curve (AUC) values, showing that MLP achieved the highest mean AUC of 0.688 (95% CI: 0.567‐0.910), followed closely by logistic regression (LR) (AUC 0.682, 95% CI: 0.462‐0.858) and eXtreme gradient boosting (XGBoost) (AUC 0.674, 95% CI: 0.386‐0.801). Gradient boosting machine (GBM) showed the lowest mean performance with an AUC of 0.639 (95% CI: 0.365‐0.696). Figure S1 illustrates the AUC values for each ML method stratified by center, highlighting some variability in model performance across different centers. For example, Center 4 showed the highest individual AUC for multi‐layer perceptron (MLP) (0.752), while LR yielded the best performance in Center 1 (0.743). These findings indicate that MLP was among the most effective local models for predicting postoperative delirium across diverse clinical settings.

### 2.3 POD Prediction Models Based on FL

Subsequently, FLMs were developed to predict POD. These models were first validated on the pooled internal validation set, as shown in Figure 2B, where the FLMs demonstrated acceptable discrimination with AUC values ranging from 0.725 to 0.726. Figure 2C shows that performance remained consistent in the external validation cohort, with AUCs between 0.700 and 0.701. Further robustness assessment with 10‐fold cross‐validation (Table S4) confirmed stable performance across folds, with AUC values ranging from 0.634 to 0.710, accompanied by balanced accuracy, sensitivity, and specificity across the iterations. Together, these results highlight the effectiveness and generalizability of the FLMs in predicting POD across diverse datasets.

### 2.4 POD FLMs Compared With LLMs and CLMs

The trained FLMs were further evaluated on the internal validation sets of each center and compared with LLMs. As shown in Table 1, FLMs constructed using different aggregation strategies including FedAvg, FedLSD, and FedProx, generally showed numerically higher AUCs than the local models across the four centers. For example, in Center 1, FLMs achieved AUCs around 0.717‐0.718, compared to 0.703 for LLM. Similar trends were observed in Centers 2, 3, and 4, where FLMs also showed higher AUCs than LLMs. Sensitivity values were also improved in several centers with FLMs, particularly in Center 3 where federated models reached sensitivities of 0.944 compared to 0.556 for the local model. DeLong's test indicated that statistically significant differences in AUC between FLMs and LLMs were observed only in Center 3, with *p* values of 0.012 for FedAvg, 0.013 for FedLSD, and 0.012 for FedProx, whereas the comparisons in the other centers were not statistically significant. Overall, these results indicate that FLMs showed a consistent trend toward numerically improved discrimination compared with local models across centers.

**TABLE 1:** Comparison of validation performance between POD local learning models and federated learning models across four centers.

Center	Model	Acc	Sensitivity	Specificity	AUC	*p*
1	Local	0.579	0.671	0.563	0.703	
	FedAvg	0.681	0.624	0.692	0.718	0.403
	FedLSD	0.682	0.631	0.692	0.717	0.413
	FedProx	0.682	0.624	0.693	0.717	0.408
2	Local	0.647	0.500	0.657	0.631	
	FedAvg	0.538	0.667	0.529	0.635	0.971
	FedLSD	0.533	0.667	0.523	0.640	0.949
	FedProx	0.538	0.667	0.529	0.634	0.978
3	Local	0.788	0.556	0.832	0.665	
	FedAvg	0.637	0.944	0.579	0.825	0.012
	FedLSD	0.646	0.944	0.589	0.820	0.013
	FedProx	0.637	0.944	0.579	0.825	0.012
4	Local	0.704	0.600	0.716	0.752	
	FedAvg	0.643	0.600	0.648	0.765	0.883
	FedLSD	0.612	0.600	0.614	0.772	0.786
	FedProx	0.643	0.600	0.648	0.766	0.883

*Note: p* values were calculated using the DeLong test to compare the AUC of each federated learning model (FedAvg, FedLSD, FedProx) against the local learning model within each center.

Finally, using the centralized data from the four centers, we constructed POD CLMs based on the MLP algorithm. Figure 2D displays the ROC curves of the CLMs, demonstrating good predictive performance with an AUC of 0.732 (95% CI: 0.693‐0.769) on the internal validation set and 0.706 (95% CI: 0.599‐0.817) on the external validation set. Subsequently, we compared the CLMs with FLMs across the same internal and external validation cohorts (Table 2). The FLMs showed comparable AUCs to the CLMs in both datasets. DeLong's test indicated no statistically significant differences between the CLMs and any FLM, with all *p‐*values greater than 0.05.

**TABLE 2:** Comparison of validation performance between POD centralized learning models and federated learning models.

Dataset	Model	Acc	Sensitivity	Specificity	AUC	AUC 95%CI	*p*
Internal Validation	Centralized	0.726	0.608	0.745	0.732	0.693–0.769	
	FedAvg	0.684	0.640	0.691	0.725	0.684–0.760	0.542
	FedLSD	0.687	0.646	0.694	0.726	0.686–0.761	0.612
	FedProx	0.685	0.640	0.692	0.725	0.684–0.760	0.544
External Validation	Centralized	0.629	0.650	0.628	0.706	0.599–0.817	
	FedAvg	0.632	0.750	0.625	0.701	0.580–0.813	0.908
	FedLSD	0.629	0.750	0.622	0.700	0.578–0.813	0.902
	FedProx	0.632	0.750	0.625	0.700	0.580–0.813	0.897

*Note: p* values were calculated using the DeLong test to compare the AUC of each federated learning model (FedAvg, FedLSD, FedProx) against the centralized learning model in both internal and external validation datasets.

Moreover, DCA curves (Figure S2) showed that both CLMs and FLMs provided similar net clinical benefits across a range of threshold probabilities in internal (Figure S2A) and external validation sets (Figure S2B), suggesting comparable clinical utility. Calibration curves and quantitative calibration metrics showed broadly similar calibration patterns across the centralized and federated models (Figure S3 and Table S5). In internal validation, the calibration intercepts were close to 0 and the slopes were close to 1. In the external validation cohort, however, the model‐specific curves were generally below the ideal calibration line, and the negative calibration intercepts indicated systematic overestimation of POD risk. Overall, the FLMs showed discrimination and clinical utility comparable to the centralized model, while exhibiting similar but imperfect external calibration.

Additional post hoc reduced‐variable benchmark analyses for POD prediction showed that the full‐variable centralized benchmark model achieved higher AUCs than both the Age + American Society of Anesthesiologists (ASA) minimal model and the preoperative‐only model in the internal validation cohort (0.732 vs. 0.612 and 0.683, respectively) and in the external validation cohort (0.706 vs. 0.568 and 0.584, respectively) (Table S6).

### 2.5 POD SHAP Analysis Results

To further explore model interpretability and inter‐center heterogeneity, we compared SHAP summary plots across the three federated strategies within each development center and additionally included the SHAP summary plot of the centralized learning model (Figure S4). Across the four centers, surgery duration was consistently among the most influential predictors of POD, and age also ranked highly in most models. The core predictors were broadly stable across the three federated aggregation strategies within each center. By contrast, inter‐center heterogeneity was mainly reflected in the relative importance of secondary features, including intraoperative medications, surgical characteristics, functional indicators, and laboratory or physiologic variables. The centralized model showed a similar overall pattern, with age and surgery duration remaining dominant predictors.

### 2.6 AKI LLMs Based on Different ML Algorithms

**FIGURE 3:**
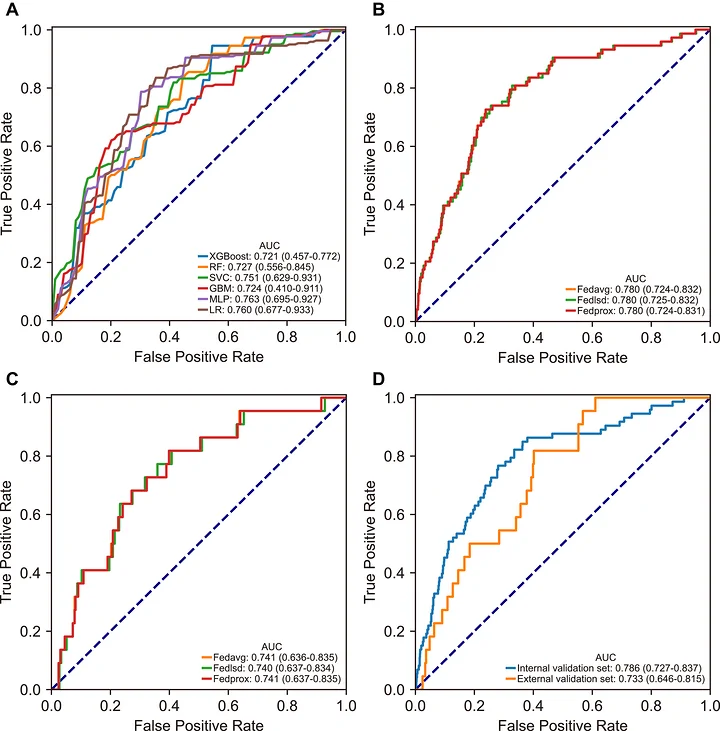
(A) Average ROC curves of AKI LLMs based on different machine learning methods across four centers. (B) Internal validation ROC curves of AKI FLMs. (C) External validation ROC curves of AKI FLMs. (D) ROC curves of AKI CLMs.

Similar to the findings in POD prediction, MLP also demonstrated strong predictive performance with an average AUC of 0.763 (Figure 3A). Model performance varied across centers, with LR achieving the highest AUC in Center 3 (0.849) and XGBoost performing well in Center 1 (0.755) (Figure S5). Overall, these results indicate that MLP is among the most effective local models for AKI prediction in diverse clinical settings.

### 2.7 AKI Prediction Models Based on FL

Subsequently, FLMs were developed to predict AKI. The models achieved AUC values of 0.780 on the pooled internal validation set and demonstrated stable performance with AUCs between 0.740 and 0.741 on the external validation set (Figure 3B,C). Further robustness assessment via 10‐fold cross‐validation (Table S7) confirmed stable performance across folds, with AUC values ranging from 0.701 to 0.817. These findings indicate stable predictive performance of the FLMs for AKI prediction across diverse datasets.

### 2.8 AKI FLMs Compared With LLMs and CLMs

The trained FLMs were evaluated on each center's internal validation set and compared with LLMs (Table 3). FLMs constructed using different aggregation strategies, including FedAvg, FedLSD, and FedProx, showed numerically higher or similar AUCs compared with local models across the four centers. However, DeLong's test indicated that these differences were not statistically significant in any center.

**TABLE 3:** Comparison of validation performance between AKI local learning models and federated learning models across four centers.

Center	Model	Acc	Sensitivity	Specificity	AUC	*p*
1	Local	0.598	0.821	0.584	0.760	
	FedAvg	0.696	0.750	0.692	0.786	0.113
	FedLSD	0.696	0.768	0.691	0.787	0.101
	FedProx	0.695	0.750	0.691	0.786	0.113
2	Local	0.614	0.778	0.606	0.683	
	FedAvg	0.587	0.889	0.571	0.728	0.667
	FedLSD	0.587	0.778	0.577	0.724	0.694
	FedProx	0.587	0.889	0.571	0.728	0.670
3	Local	0.708	0.750	0.706	0.787	
	FedAvg	0.699	0.750	0.697	0.796	0.912
	FedLSD	0.690	0.750	0.688	0.796	0.883
	FedProx	0.690	0.750	0.688	0.794	0.912
4	Local	0.735	0.750	0.734	0.822	
	FedAvg	0.776	0.750	0.777	0.827	0.906
	FedLSD	0.776	0.750	0.777	0.827	0.906
	FedProx	0.776	0.750	0.777	0.827	0.906

*Note: p* values were calculated using the DeLong test to compare the AUC of each federated learning model (FedAvg, FedLSD, FedProx) against the local learning model within each center.

Based on centralized data from four centers, AKI CLMs were constructed using the MLP algorithm, achieving AUCs of 0.786 (95% CI: 0.727‐0.837) internally and 0.733 (95% CI: 0.646‐0.815) externally (Figure 3D). Compared with FLMs, no significant differences in AUC were observed (all *p* > 0.05) (Table 4). DCA showed similar net clinical benefits between the centralized and federated models (Figure S6). The calibration curves and quantitative metrics also showed broadly similar patterns across models, although the calibration slopes indicated deviation from ideal calibration (Figure S7 and Table S8). The calibration intercepts were close to 0, whereas the calibration slopes were below 1, with broadly similar Brier scores across the centralized and federated models.

**TABLE 4:** Comparison of validation performance between AKI centralized learning models and federated learning models.

Dataset	Model	Acc	Sensitivity	Specificity	AUC	AUC 95%CI	*p*
Internal Validation	Centralized	0.641	0.849	0.630	0.786	0.727–0.837	
	FedAvg	0.687	0.781	0.682	0.780	0.724–0.832	0.736
	FedLSD	0.686	0.767	0.682	0.780	0.725–0.832	0.729
	FedProx	0.686	0.767	0.681	0.780	0.724–0.831	0.735
External Validation	Centralized	0.609	0.773	0.598	0.733	0.646–0.815	
	FedAvg	0.700	0.682	0.701	0.741	0.636–0.835	0.792
	FedLSD	0.703	0.682	0.704	0.740	0.637–0.834	0.820
	FedProx	0.700	0.682	0.701	0.741	0.637–0.835	0.791

*Note: p* values were calculated using the DeLong test to compare the AUC of each federated learning model (FedAvg, FedLSD, FedProx) against the centralized learning model in both internal and external validation datasets.

Additional post hoc reduced‐variable benchmark analyses for AKI prediction showed that the full‐variable centralized benchmark model achieved higher AUCs than both the Age + ASA minimal model and the preoperative‐only model in the internal validation cohort (0.786 vs. 0.667 and 0.721, respectively) and in the external validation cohort (0.733 vs. 0.560 and 0.586, respectively) (Table S9).

### 2.9 AKI SHAP Analysis Results

Compared with POD, the SHAP patterns for AKI suggest a different feature structure. Across the four development centers, surgery duration, surgical department, ASA classification, age, and type of surgery were repeatedly identified as important predictors. The main SHAP patterns were broadly similar across the three federated aggregation strategies within each center, whereas inter‐center variation was more apparent in the ranking of secondary features, including hypertension, postoperative analgesia, surgical site, MET score, serum albumin, malignant tumor, and medication‐related variables (Figure S8). The centralized model showed a similar overall pattern, with surgery duration, ASA classification, age, type of surgery, and surgical department ranking highest.

## 3 Discussion

Using multicenter data from four development centers, we developed FLMs to predict postoperative delirium and acute kidney injury in older surgical patients. Our principal finding is that FLMs achieved predictive performance comparable to CLMs for both outcomes in a simulated decentralized training setting without direct pooling of raw patient data. More importantly, the full‐variable models showed substantially better external discrimination than the Age + ASA and preoperative‐only benchmark models, indicating meaningful incremental value from broader perioperative information. Compared with LLMs, FLMs showed a consistent trend toward numerically better or comparable discrimination across centers for both outcomes, with a statistically significant improvement for POD prediction in Center 3 by DeLong testing. Overall, our findings support federated learning as a feasible privacy‐preserving strategy for multicenter perioperative prediction modeling.

POD and AKI significantly affect the recovery and quality of life of older patients. Accurate prediction of these complications may facilitate targeted preoperative intervention and perioperative monitoring, thereby potentially reducing their occurrence. Previous researchers, such as Rössler et al. [32] and our team, have shown that ML models can achieve high accuracy in predicting these risks, offering valuable risk stratification for older patients [33]. In a single‐center retrospective cohort study of 111,888 operations, Xue et al. used five ML algorithms to predict postoperative complications, achieving AUCs of 0.848 for AKI and 0.762 for delirium [34]. Although these studies demonstrated the potential of ML in predicting postoperative complications, they also highlighted several limitations inherent to traditional ML approaches. One major drawback is the need for large centralized datasets, which can be difficult to compile owing to data privacy concerns and regulatory restrictions [35]. Most large datasets come from a single center, leading to potential biases. Moreover, the variability in data collection methods across institutions can result in inconsistent data quality, further complicating model development and reducing model generalizability [36]. These challenges underscore the need for innovative approaches that can leverage the collective power of diverse datasets while maintaining data privacy and security.

FL offers key advantages over centralized methods, including enhanced data privacy by keeping patient data local [37, 38]. It also improves model robustness by using diverse data sources, enhancing generalizability and accuracy [39]. Building on these advantages, FL has been successfully applied in various disease prediction tasks, demonstrating its practical potential in real‐world scenarios. Several studies have demonstrated the application of FL in disease prediction. Danek et al. used multi‐omics data from two datasets to predict the onset of Parkinson's disease. The best FL model achieved an AUC‐PR of 87.6%, with a performance difference of less than 2% compared to the CLM [30]. Sheller et al. applied FL to the BraTS 2018 dataset to build a brain tumor segmentation model. The best FLM achieved a Dice coefficient of 0.852, only 0.01 lower than the CLM (0.862). FL not only protects data privacy but also outperforms other collaborative methods (e.g., IIL and CIIL), demonstrating stability even with imbalanced multi‐institutional data distributions [40].

Additionally, FL enables smaller institutions with limited data to access high‐quality models, addressing data imbalance and scarcity effectively [41]. This practical advantage may be particularly important for smaller medical centers or rare‐case settings, where local data are often insufficient for independent model development [27]. In brain tumor segmentation, FL performed well even with as few as six samples from individual institutions [42]. In our study, where some centers provided only a few hundred samples and others thousands, FL showed competitive performance despite marked sample‐size imbalance, supporting its potential adaptability in multicenter settings. This imbalance is common in real‐world multicenter clinical practice and may make our setting more representative of realistic deployment scenarios than an idealized balanced distribution. Under such non‐IID conditions, sample‐size imbalance and center‐specific data distributions may affect centralized and federated learning through different mechanisms. Centralized learning may be influenced by the majority distribution of the pooled data, whereas federated learning may be affected by heterogeneous local updates and sample‐size‐weighted aggregation. In the present study, FLMs nevertheless achieved performance broadly comparable to CLMs under the observed degree of multicenter heterogeneity.

The lower POD incidence in the external validation cohort (5.7% vs. 13.8%), together with the significant differences in baseline and perioperative characteristics between the development and external validation cohorts, suggests the presence of domain shift. These significant between‐dataset differences may partly explain the modest decline in external validation performance, particularly for AKI prediction. This is likely attributable to differences in patient case mix, perioperative management, and institutional practice patterns. In particular, the external validation center is a specialized neuroscience institution, where non‐neurosurgical older patients may represent a more selected population with a different perioperative risk profile from those in the development cohort. Consistent with the lower POD incidence in the external cohort, the negative calibration intercepts indicated systematic overestimation of absolute POD risk, although the calibration patterns remained broadly similar across the centralized and federated models. The relatively small sample size and low POD incidence of the external validation cohort may also have reduced the precision and robustness of the external performance estimates. The limited number of POD events likely increased statistical uncertainty, as reflected by the relatively wide 95% confidence intervals in the external validation results. Therefore, the exact magnitude of model performance in this cohort should be interpreted with appropriate caution. Despite these limitations, the federated models maintained clinically meaningful discrimination in the external cohort, suggesting transportability of their risk‐ranking performance, although local recalibration may be required before clinical implementation.

Post hoc sensitivity and reduced‐variable benchmark analyses further helped clarify the clinical value of the proposed framework. Specifically, the Age + ASA minimal models showed substantially lower discrimination than the full‐variable centralized benchmark models for both POD and AKI, particularly in external validation. The preoperative‐only models retained some predictive information but also performed worse than the full‐variable models. These findings suggest that the observed model performance was not explained by simple baseline clinical characteristics alone and that intraoperative and perioperative variables contributed meaningful incremental predictive value. Accordingly, the practical value of federated learning in this setting lies not merely in marginal gains in AUC, but in its ability to approximate centralized‐model performance without direct data sharing while outperforming simpler baseline models in a multicenter privacy‐constrained environment.

An additional strength of our study is that the SHAP analysis provided insight not only into globally important predictors but also into center‐specific heterogeneity across both POD and AKI models. For POD, age and surgery duration were stable shared predictors across federated and centralized models, whereas for AKI, surgery duration, surgical department, and ASA classification were more prominent. Beyond these common signals, the relative importance of secondary features varied across institutions and generally involved medication‐related variables, physiological or laboratory indicators, and surgery‐related characteristics. From a perioperative perspective, these differences likely reflect variation in patient case mix, operative spectrum, anesthetic and analgesic pathways, and local documentation practices. Although these SHAP patterns should be interpreted as model‐level explanations rather than causal effects, they suggest that the federated models captured both shared risk factors across centers and center‐specific variation under multicenter heterogeneity.

Despite its rigorous design, several limitations remain. First, although we included five centers and applied multiple modeling strategies, model performance remained limited, likely due to suboptimal center‐level performance, distributional differences between training and external validation cohorts, and limited perioperative feature granularity. FL enables privacy‐preserving multicenter collaboration but cannot overcome limitations in data quality or feature completeness. Future studies with larger samples and more detailed perioperative variables may improve performance. Second, the federated learning framework was evaluated as a retrospective simulation rather than a live deployed inter‐hospital network. Centralized feature selection in this simulation does not fully reflect an end‐to‐end federated workflow. In a prospective federated deployment, candidate variables could be harmonized using a prespecified common data dictionary, with locally derived feature statistics or importance scores combined through privacy‐preserving aggregation. The internal validation results should be interpreted as development‐stage evaluations because these datasets informed architecture selection. Therefore, practical implementation issues such as communication latency, system connectivity, asynchronous updating, and site‐level computational variability were not assessed in this study. Third, data heterogeneity could impair the generalizability of federated models, as data in FL frameworks are often non‐IID [43]. Although FedLSD was included as an aggregation strategy designed to address heterogeneity, the imbalanced sample distribution across centers remains a structural limitation but is also common in real‐world clinical practice. This setting realistically reflects current clinical conditions and may have greater practical applicability than an idealized balanced distribution [44]. Future studies should include more balanced multicenter samples to ensure more equitable and robust contributions.

In conclusion, FL holds substantial potential for developing privacy‐preserving prediction models for postoperative complications in older patients. By utilizing decentralized data without direct sharing of raw patient information, it may support collaborative model development across healthcare institutions. This approach may facilitate early risk stratification for complications such as POD and AKI and help guide targeted perioperative monitoring and resource allocation.

## 4 Materials and Methods

### 4.1 Study Design

This multicenter study used data from four centers in the development cohort. Federated learning was evaluated in a retrospective simulation setting, with the data partitioned according to center of origin. Initially, six different ML algorithms were trained and evaluated independently at each center using the corresponding training and internal validation sets. Their performance across the four centers was considered collectively to select a single common architecture for subsequent modeling, rather than selecting a different algorithm for each center. Using the selected MLP architecture, FLMs were then constructed and evaluated using 10‐fold cross‐validation. Subsequently, the trained FLMs were evaluated on the internal validation sets from each of the four centers. Because these internal validation sets informed architecture selection, the corresponding results were considered development‐stage evaluations, whereas the external validation cohort was completely held out for independent evaluation. Their performance was compared with the corresponding LLMs on the same validation sets to assess the advantages and limitations of FL relative to single‐center local models. Finally, CLMs were built by pooling data from all four centers. The predictive performance of the CLMs and FLMs was compared on both internal and external validation cohorts to evaluate model generalizability and practical applicability. This study design allowed us to leverage multicenter data while simulating privacy‐preserving collaboration and provided a comprehensive evaluation of the potential of FL in predicting postoperative complications in older patients.

### 4.2 Study Participants

Clinical data from older surgical patients (excluding cardiac and neurosurgical patients) were collected from five centers between April 2020 and April 2022. The development dataset included four centers: Chinese PLA General Hospital; The Second Xiangya Hospital of Central South University; Union Hospital, Tongji Medical College, Huazhong University of Science and Technology; and The Affiliated Hospital of Guizhou Medical University. Data from Beijing Tiantan Hospital, Capital Medical University, were used as the external validation dataset. This center was designated a priori before model development because it had the smallest eligible cohort, allowing the four larger institutional cohorts to be retained for model development.

The inclusion criteria were as follows: (1) age ≥ 65 years; (2) patients undergoing non‐cardiac, non‐neurosurgical surgery; and (3) surgery performed between April 2020 and April 2022. Patients were excluded if they underwent gastrointestinal endoscopy, had missing procedure names, had missing outcome data, had abnormal data values identified during data quality control, or had > 40% missing data across overall variables. In addition, variables with > 20% missing data across patients were excluded from model development.

### 4.3 Dataset Acquisition

Data regarding the preoperative and intraoperative parameters were collected. The basic characteristics of the patients obtained preoperatively from face‐to‐face interviews included age, sex, body mass index (BMI), education level, smoking, and alcohol consumption statuses; history of coronary heart disease, hypertension, diabetes, anemia, liver dysfunction, renal dysfunction, and malignant tumors; and metabolic equivalent (MET) score. The preoperative laboratory tests included measurements of the white blood cell (WBC) count and the hemoglobin, alanine aminotransferase (ALT), aspartate aminotransferase (AST), glucose, serum albumin, urea, serum creatinine, potassium (K), sodium (Na), and calcium (Ca) levels. Use of analgesics within 24 h before surgery was also recorded. The surgical department for each patient was noted. The intraoperative data included the surgery type, surgical site, malignant tumor surgery, surgical grade, surgical incision classification, number of surgical incisions, number of drainage tubes, estimated blood loss, surgical duration, urine volume, crystalloid volume, ASA classification, and anesthesia method. Intraoperative medication variables included the use of nonsteroidal anti‐inflammatory drugs (NSAIDs), benzodiazepines, ketamine, opioid analgesics, local anesthetics, ondansetron, tropisetron, dexamethasone, methylprednisolone, flurbiprofen, parecoxib, ketorolac, and opioid antagonists. Postoperative data included postoperative analgesia pump use, POD incidence, and AKI incidence. The postoperative analgesia variable indicated whether a postoperative analgesia pump was used or initiated at the end of surgery and was recorded before POD and AKI outcome ascertainment.

### 4.4 Definitions of Outcomes

POD was defined as the occurrence of delirium within 7 days after surgery, assessed using the 3‐Minute Diagnostic Interview for CAM‐defined Delirium (3D‐CAM).

AKI was defined as the occurrence of AKI within 7 days after surgery according to the Kidney Disease: Improving Global Outcomes guidelines. AKI was characterized by either an absolute rise in serum creatinine (sCr) levels ≥ 26.5 µmol/L within 48 h after surgery or a 1.5‐fold increase from the preoperative baseline sCr level within 7 days after surgery. The preoperative baseline sCr level was determined using the most recent measurement taken within 30 days before surgery.

### 4.5 Model Building Strategy

#### 4.5.1 Data Preprocessing

After screening based on the inclusion and exclusion criteria, we used 80% of the data from each center for training and the remaining 20% for internal validation. For CLMs, the training sets from all four centers were combined into a single centralized training set, whereas the internal validation sets from the four centers were pooled into a single internal validation set. All subsequent data‐driven preprocessing steps were fitted using the training data only and then applied to the corresponding internal validation sets and the external validation cohort. We applied mean imputation for numerical variables and mode imputation for categorical variables as preliminary fill‐ins using the training data only. Subsequently, we trained a random forest (RF) regression model using the preliminarily filled training dataset to predict and re‐impute the missing values. After addressing the missing values, we standardized the numerical variables by removing the mean and scaling to unit variance, with scaling parameters estimated from the training data and then applied to the validation data. Moreover, we transformed the categorical variables into one‐hot encoding using an encoding scheme defined from the training data. Feature selection was conducted separately for the POD and AKI prediction tasks using only the pooled training data from the four development centers. Neither the center‐specific internal validation sets nor the external validation cohort was involved in this process. Feature importance was calculated on the corresponding pooled training dataset, and features with an importance value below 0.001 were removed. The resulting feature subset was then applied uniformly across all participating sites to ensure a consistent feature space before federated training. This centralized feature‐selection procedure was used to define a common feature space in the present retrospective simulation.

Owing to class imbalance, resampling was performed only on the training data. We first used the Synthetic Minority Over‐sampling Technique (SMOTE) to generate more minority class samples until a certain imbalance ratio was reached, which is the number of positive samples over negative samples. We then employed the edited nearest neighbor to remove noisy samples from the boundaries. Specifically, for the POD and AKI tasks, we set the imbalance ratio to 0.4:1.0.

#### 4.5.2 Local Learning Models for Each Center

For comparison, as a baseline, we employed six ML models for local datasets to evaluate their performance in predicting postoperative complications in older adults. These models included MLP, LR, XGBoost, support vector classification (SVC), RF, and GBM.

To evaluate model performance across centers, ROC curves and AUC values were first calculated separately for each center using the corresponding predicted probabilities and true labels. The center‐specific ROC curves were then interpolated onto a common false positive rate grid and averaged to generate a mean ROC curve for each model across centers, from which the mean AUC was derived. Confidence intervals for the AUC were estimated using bootstrap resampling. The resulting average ROC curves and corresponding AUC values with confidence intervals were displayed in the same coordinate system to facilitate intuitive comparison of model performance across different methods.

#### 4.5.3 FL Among Multiple Centers

To address data privacy concerns across centers while effectively training the models, we employed the following FL algorithms:

- FedAvg [45]: FedAvg updates the global model by training local models on different clients and aggregating their parameters. It enhances the generalization performance of the model while protecting data privacy.
- FedProx [46]: FedProx includes a regularization term during local training to constrain the differences between the local and global models, thereby improving the model stability and convergence speed.
- FedLSD [47]: FedLSD leverages global knowledge to guide local data and preserve global knowledge during local training to mitigate data heterogeneity issues. It improves model convergence without accessing client data.

The federated learning process was simulated by partitioning the development dataset according to center of origin and treating each partition as an individual client. Local model training and parameter aggregation were performed to emulate federated updating across centers. After a predefined number of local epochs, model parameters from each center‐specific partition were aggregated into a global model, and this process was repeated for multiple communication rounds. This design allowed us to evaluate the feasibility and predictive performance of federated learning in a multicenter setting without direct pooling of raw data.

#### 4.5.4 Centralized Learning Models

To provide a reference benchmark for federated learning, CLMs were constructed by pooling the training data from the four development centers. The same preprocessing procedure, feature set, and MLP architecture used for federated learning were applied to the CLMs. Model performance was evaluated on the pooled internal validation cohort from the four development centers and on the independent external validation cohort. The CLMs were used as full‐variable reference models in the main performance comparison and in the sensitivity analyses.

Additional post hoc sensitivity and reduced‐variable benchmark analyses were performed separately for POD and AKI prediction using the corresponding centralized benchmark models. These reduced‐variable models were implemented within the centralized‐learning framework to quantify the incremental predictive value of the full perioperative feature set without introducing additional variability from federated aggregation strategies. For both POD and AKI prediction, we constructed two reduced‐variable models: a minimal model including only age and ASA classification, and a preoperative‐only model including presurgical variables only, such as demographics, comorbidities, BMI, ASA classification, preoperative MET score, and surgical department/site.

#### 4.5.5 Evaluation Metrics

We comprehensively evaluated the performance of the ML models using several key metrics, including the AUC, accuracy, sensitivity (recall), and specificity, to assess their classification effectiveness and generalization capability from different perspectives. We also conducted DCA and calibration curve analysis of the internal and external validation datasets.

To obtain calibrated probabilities, beta calibration was fitted separately for each outcome and model using only the original, non‐resampled development training data. The resulting calibration parameters were fixed and applied without refitting to the internal and external validation cohorts. Calibration curves, calibration intercepts, calibration slopes, and Brier scores were calculated using these fixed beta‐calibrated probabilities. An intercept of 0 and a slope of 1 indicate ideal calibration, whereas a lower Brier score indicates lower overall probabilistic prediction error.

As the complexity of ML models increases, relying solely on evaluation metrics to measure the model performance is insufficient. Understanding the decision‐making process of models is crucial for ensuring their reliability and interpretability. Therefore, we introduced SHAP to support model interpretability [48]. By visualizing SHAP values, we aimed to interpret model behavior, identify influential predictors, and compare shared versus center‐specific feature importance patterns across centers and outcomes.

### 4.6 Statistical Analysis

All statistical significance tests were two‐sided, with significance defined as *p* < 0.05. Baseline table statistics were performed using R version 4.3.2 (R Foundation for Statistical Computing, Vienna, Austria). The ML models were constructed using Python version 3.8.13 (Python Software Foundation, Wilmington, DE, USA).

## Author Contributions

The study conception and design were collaboratively developed by Wei‐dong Mi, Yi‐qiang Chen, and Jiang‐bei Cao. Administrative support was provided by Yi‐qiang Chen and Jiang‐bei Cao. Data collection was conducted by Qian Wang, Xiao‐dong Wu, Ao Li, Jing‐sheng Lou, Hao Li, Yan‐hong Liu, Jun‐mei Xu, Di‐fen Wang, Qing‐ping Wu, and Yu‐ming Peng. Data analysis and interpretation were performed by Qian Wang, Yu‐xiang Song, Xiao‐dong Yang, and Jing‐wei Zhang. The manuscript was written by Qian Wang, Yu‐xiang Song, Xiao‐dong Yang, and Jing‐wei Zhang. The manuscript was revised by Qian Wang, Yu‐xiang Song, Jing‐wei Zhang, and Rui‐zhe Sun. Wei‐dong Mi was responsible for the overall content. All authors have read and approved the final manuscript.

## Funding

This study was supported by the National Key Research and Development Program of China (grant number: 2018YFC2001901) and the Beijing Natural Science Foundation (grant number: L222100).

## Ethics Statement

The study was registered with the Chinese Clinical Trial Registry (ChiCTR2400089362). This retrospective study was conducted in compliance with the Declaration of Helsinki and was approved by the Ethics Committee of the First Medical Center of the Chinese PLA General Hospital (Approval Number: S2024‐324‐01). Given the retrospective nature of the study, the requirement for informed consent was waived by the Ethics Committee. All patient data were de‐identified and anonymized prior to analysis.

## Conflicts of Interest

The authors declare no conflicts of interest.

## Supporting information




**Figure S1**: ROC curves of POD local learning models developed using different machine learning methods across four centers. (A) Chinese PLA General Hospital; (B) The Second Xiangya Hospital of Central South University; (C) Union Hospital, Tongji Medical College, Huazhong University of Science and Technology; (D) The Affiliated Hospital of Guizhou Medical University.
**Figure S2**: Decision curve analysis for all POD prediction models. (A) Internal Validation; (B) External Validation.
**Figure S3**: Calibration curve for all prediction models of POD. (A) Internal Validation; (B) External Validation.
**Figure S4**: Feature‐specific SHAP values of federated and centralized prediction models for POD across four development centers. (A‐C) Chinese PLA General Hospital; (D‐F) The Second Xiangya Hospital of Central South University; (G‐I) Union Hospital, Tongji Medical College, Huazhong University of Science and Technology; and (J‐L) The Affiliated Hospital of Guizhou Medical University. Within each center, the three panels correspond to the federated aggregation strategies FedAvg, FedLSD, and FedProx, respectively. (M) SHAP summary plot of the centralized learning model (CLM) trained on the pooled data from all four development centers. The x‐axis shows SHAP values, which represent the contribution of each feature to the model output, and the color scale indicates feature values from low to high. All medication‐related variables shown in the figure refer to intraoperative medications.
**Figure S5**: ROC curves of AKI local learning models developed using different machine learning methods across four centers. (A) Chinese PLA General Hospital; (B) The Second Xiangya Hospital of Central South University; (C) Union Hospital, Tongji Medical College, Huazhong University of Science and Technology; (D) The Affiliated Hospital of Guizhou Medical University.
**Figure S6**: Decision curve analysis for all AKI prediction models. (A) Internal Validation; (B) External Validation.
**Figure S7**: Calibration curve for all prediction models of AKI. (A) Internal Validation; (B) External Validation.
**Figure S8**: Feature‐specific SHAP values of federated and centralized prediction models for AKI across four development centers. (A‐C) Chinese PLA General Hospital; (D‐F) The Second Xiangya Hospital of Central South University; (G‐I) Union Hospital, Tongji Medical College, Huazhong University of Science and Technology; and (J‐L) The Affiliated Hospital of Guizhou Medical University. Within each center, the three panels correspond to the federated aggregation strategies FedAvg, FedLSD, and FedProx, respectively. (M) SHAP summary plot of the centralized learning model (CLM) trained on the pooled data from all four development centers. The x‐axis shows SHAP values, which represent the contribution of each feature to the model output, and the color scale indicates feature values from low to high. All medication‐related variables shown in the figure refer to intraoperative medications.
**Table S1**: Distribution of variables across the five participating centers.
**Table S2**: Participant characteristics when building POD model.
**Table S3**: Participant characteristics when building AKI model.
**Table S4**: Performance of POD Federated Learning Models in 10‐fold Cross‐validation.
**Table S5**: Calibration performance of centralized and federated learning models for POD.
**Table S6**: Validation performance of sensitivity analysis models for POD prediction.
**Table S7**: Performance of AKI Federated Learning Models in 10‐fold Cross‐validation.
**Table S8**: Calibration performance of centralized and federated learning models for AKI.
**Table S9**: Validation performance of sensitivity analysis models for AKI prediction.

## Data Availability

The datasets generated and/or analyzed during the current study are not publicly available because of institutional and patient privacy restrictions but are available from the corresponding author on reasonable request, subject to relevant approvals. The source code used to develop, train, and evaluate the FLMs in this study is publicly available at https://anonymous.4open.science/r/FL‐POD‐AKI/. The repository includes scripts for data preprocessing, model implementation, performance evaluation, and SHAP‐based interpretability analysis. Instructions for reproducing the results are provided in the README file.
