# Supplementary material for: Multicenter Privacy‐Preserving Federated Models for Predicting Postoperative Delirium and Acute Kidney Injury in Older Patients
Source: MedComm (2020). 2026 Aug 22;7(9):e70944. doi: 10.1002/mco2.70944 (PMC13499364; doi:10.1002/mco2.70944)
Supplement: Supplementary file 1 [file MCO2-7-e70944-s001.docx]

**Supplementary Materials**

**Multicenter privacy-preserving federated models for predicting postoperative delirium and acute kidney injury in older patients**

**Short Title:** Federated Learning for Postoperative Prediction

Qian Wang, MS^1,2,3#^, Yu-xiang Song, MD^1,3,4#^, Xiao-dong Yang, PhD^5#^, Jing-wei Zhang, MS^5,6#^, Rui-zhe Sun, MD^5^, Xiao-dong Wu, MD^1^, Ao Li, MD^1^, Jing-sheng Lou, MD^1^, Hao Li, MD^1^, Yan-hong Liu, MD^1^, Jun-mei Xu, MD^7^, Di-fen Wang, MD^8^, Qing-ping Wu, MD^9^, Yu-ming Peng, MD^10^, Yi-qiang Chen, PhD^5*^, Jiang-bei Cao, MD^1,3,4*^, Wei-dong Mi, MD^1,3,4*^

^#^These authors contributed equally to this work.

^1^ Department of Anaesthesiology, The First Medical Center of Chinese PLA General Hospital, Beijing 100853, China.

^2^ Chinese PLA Medical School, Beijing 100853, China.

^3^ Beijing Key Laboratory of Perioperative Multimodal Digital Intelligence-Integrated Diagnosis and Treatment, Beijing 100853, China.

^4^ National Clinical Research Center for Geriatric Diseases, People's Liberation Army General Hospital, Beijing 100853, China.

^5^ Institute of Computing Technology, Chinese Academy of Sciences, Beijing 100190, China.

^6^ Medical School, Tianjin University, Tianjin 300072, China.

^7^ Department of Anaesthesiology, The Second Xiangya Hospital of Central South University, Changsha 410012, China.

^8^ Department of Intensive Care Medicine, The Affiliated Hospital of Guizhou Medical University, Guiyang 550004, China.

^9^ Department of Anaesthesiology, Union Hospital, Tongji Medical College, Huazhong University of Science and Technology, Wuhan 430022, China.

^10^ Department of Anaesthesiology, Beijing Tiantan Hospital, Capital Medical University, Beijing 100070, China.

* Corresponding authors:

Wei-dong Mi, Department of Anaesthesiology, The First Medical Center of Chinese PLA General Hospital, Beijing 100853, China. Email: wwdd1962@aliyun.com

Jiang-bei Cao, Department of Anaesthesiology, The First Medical Center of Chinese PLA General Hospital, Beijing 100853, China. Email: [caojiangbei@301hospital.com.cn](mailto:caojiangbei@301hospital.com.cn)

Yi-qiang Chen, Institute of Computing Technology, Chinese Academy of Sciences, Beijing 100190, China. Email: yqchen@ict.ac.cn

**Supplementary Materials**

**Figure S1.** ROC curves of POD local learning models developed using different machine learning methods across four centers. (A) Chinese PLA General Hospital; (B) The Second Xiangya Hospital of Central South University; (C) Union Hospital, Tongji Medical College, Huazhong University of Science and Technology; (D) The Affiliated Hospital of Guizhou Medical University.
**Figure S2.** Decision curve analysis for all POD prediction models. (A) Internal Validation; (B) External Validation.

**Figure S3.** Calibration curve for all prediction models of POD. (A) Internal Validation; (B) External Validation.

**Figure S4.** Feature-specific SHAP values of federated and centralized prediction models for POD across four development centers. (A-C) Chinese PLA General Hospital; (D-F) The Second Xiangya Hospital of Central South University; (G-I) Union Hospital, Tongji Medical College, Huazhong University of Science and Technology; and (J-L) The Affiliated Hospital of Guizhou Medical University. Within each center, the three panels correspond to the federated aggregation strategies FedAvg, FedLSD, and FedProx, respectively. (M) SHAP summary plot of the centralized learning model (CLM) trained on the pooled data from all four development centers. The x-axis shows SHAP values, which represent the contribution of each feature to the model output, and the color scale indicates feature values from low to high. All medication-related variables shown in the figure refer to intraoperative medications.

**Figure S5.** ROC curves of AKI local learning models developed using different machine learning methods across four centers. (A) Chinese PLA General Hospital; (B) The Second Xiangya Hospital of Central South University; (C) Union Hospital, Tongji Medical College, Huazhong University of Science and Technology; (D) The Affiliated Hospital of Guizhou Medical University.
**Figure S6.** Decision curve analysis for all AKI prediction models. (A) Internal Validation; (B) External Validation.

**Figure S7.** Calibration curve for all prediction models of AKI. (A) Internal Validation; (B) External Validation.

**Figure S8.** Feature-specific SHAP values of federated and centralized prediction models for AKI across four development centers. (A-C) Chinese PLA General Hospital; (D-F) The Second Xiangya Hospital of Central South University; (G-I) Union Hospital, Tongji Medical College, Huazhong University of Science and Technology; and (J-L) The Affiliated Hospital of Guizhou Medical University. Within each center, the three panels correspond to the federated aggregation strategies FedAvg, FedLSD, and FedProx, respectively. (M) SHAP summary plot of the centralized learning model (CLM) trained on the pooled data from all four development centers. The x-axis shows SHAP values, which represent the contribution of each feature to the model output, and the color scale indicates feature values from low to high. All medication-related variables shown in the figure refer to intraoperative medications.

**Table S1.** Distribution of variables across the five participating centers.

**Table S2.** Participant characteristics when building POD model

**Table S3.** Participant characteristics when building AKI model

**Table S4.** Performance of POD Federated Learning Models in 10-fold Cross-validation.

**Table S5.** Calibration performance of centralized and federated learning models for POD.

**Table S6.** Validation performance of sensitivity analysis models for POD prediction.

**Table S7.** Performance of AKI Federated Learning Models in 10-fold Cross-validation.

**Table S8.** Calibration performance of centralized and federated learning models for AKI.

**Table S9.** Validation performance of sensitivity analysis models for AKI prediction.

**Figure S1.** ROC curves of POD local learning models developed using different machine learning methods across four centers. (A) Chinese PLA General Hospital; (B) The Second Xiangya Hospital of Central South University; (C) Union Hospital, Tongji Medical College, Huazhong University of Science and Technology; (D) The Affiliated Hospital of Guizhou Medical University.

**Figure S2.** Decision curve analysis for all POD prediction models. (A) Internal Validation; (B) External Validation.

**Figure S3.** Calibration curve for all prediction models of POD. (A) Internal Validation; (B) External Validation.

**Figure S4.** Feature-specific SHAP values of federated and centralized prediction models for POD across four development centers. (A-C) Chinese PLA General Hospital; (D-F) The Second Xiangya Hospital of Central South University; (G-I) Union Hospital, Tongji Medical College, Huazhong University of Science and Technology; and (J-L) The Affiliated Hospital of Guizhou Medical University. Within each center, the three panels correspond to the federated aggregation strategies FedAvg, FedLSD, and FedProx, respectively. (M) SHAP summary plot of the centralized learning model (CLM) trained on the pooled data from all four development centers. The x-axis shows SHAP values, which represent the contribution of each feature to the model output, and the color scale indicates feature values from low to high. All medication-related variables shown in the figure refer to intraoperative medications.

**Figure S5.** ROC curves of AKI local learning models developed using different machine learning methods across four centers. (A) Chinese PLA General Hospital; (B) The Second Xiangya Hospital of Central South University; (C) Union Hospital, Tongji Medical College, Huazhong University of Science and Technology; (D) The Affiliated Hospital of Guizhou Medical University.

**Figure S6.** Decision curve analysis for all AKI prediction models. (A) Internal Validation; (B) External Validation.

**Figure S7.** Calibration curve for all prediction models of AKI. (A) Internal Validation; (B) External Validation.

**Figure S8.** Feature-specific SHAP values of federated and centralized prediction models for AKI across four development centers. (A-C) Chinese PLA General Hospital; (D-F) The Second Xiangya Hospital of Central South University; (G-I) Union Hospital, Tongji Medical College, Huazhong University of Science and Technology; and (J-L) The Affiliated Hospital of Guizhou Medical University. Within each center, the three panels correspond to the federated aggregation strategies FedAvg, FedLSD, and FedProx, respectively. (M) SHAP summary plot of the centralized learning model (CLM) trained on the pooled data from all four development centers. The x-axis shows SHAP values, which represent the contribution of each feature to the model output, and the color scale indicates feature values from low to high. All medication-related variables shown in the figure refer to intraoperative medications.

**Table S1. Distribution of variables across the five participating centers.**

| **Variable** | **Chinese PLA General Hospital** | **The Second Xiangya Hospital of Central South University** | **Union Hospital, Tongji Medical College, Huazhong University of Science and Technology** | **The Affiliated Hospital of Guizhou Medical University** | **Beijing Tiantan Hospital, Capital Medical University** | **P value** | **CV (%)** | **Missing (%)** |
| --- | --- | --- | --- | --- | --- | --- | --- | --- |
| N | 4894 | 920 | 562 | 487 | 353 |  |  |  |
| Sex, n (%) |  |  |  |  |  | <0.001 | 8.6% | 0.0% |
| Female | 2360 (48.2%) | 377 (41.0%) | 215 (38.3%) | 220 (45.2%) | 178 (50.4%) |  |  |  |
| Male | 2534 (51.8%) | 543 (59.0%) | 347 (61.7%) | 267 (54.8%) | 175 (49.6%) |  |  |  |
| Age, years (Median (IQR)) | 71.00 (68.00, 75.00) | 71.00 (68.00, 74.00) | 70.00 (67.25, 73.00) | 72.00 (70.00, 76.00) | 71.00 (67.00, 75.00) | <0.001 | 1.0% | 0.0% |
| BMI, kg/m² (Median (IQR)) | 24.50 (22.50, 26.80) | 22.80 (20.90, 25.00) | 25.15 (24.23, 26.07) | 23.00 (20.92, 25.35) | 24.40 (22.50, 27.00) | <0.001 | 4.3% | 9.6% |
| Educational Level, n (%) |  |  |  |  |  | <0.001 | 30.3% | 9.5% |
| Below primary school | 356 (7.3%) | 59 (6.4%) | 50 (8.9%) | 102 (20.9%) | 22 (6.2%) |  |  |  |
| Primary school | 810 (16.6%) | 444 (48.3%) | 105 (18.7%) | 121 (24.8%) | 55 (15.6%) |  |  |  |
| Junior high school | 1194 (24.4%) | 164 (17.8%) | 169 (30.1%) | 101 (20.7%) | 116 (32.9%) |  |  |  |
| High school (including technical secondary school) | 852 (17.4%) | 112 (12.2%) | 107 (19.0%) | 91 (18.7%) | 78 (22.1%) |  |  |  |
| College, junior college | 1043 (21.3%) | 102 (11.1%) | 116 (20.6%) | 65 (13.3%) | 59 (16.7%) |  |  |  |
| Postgraduate or above | 30 (0.6%) | 2 (0.2%) | 2 (0.4%) | 1 (0.2%) | 4 (1.1%) |  |  |  |
| Smoke, n (%) |  |  |  |  |  | 0.071 | 4.4% | 0.6% |
| No | 3611 (73.8%) | 662 (72.0%) | 408 (72.6%) | 332 (68.2%) | 272 (77.1%) |  |  |  |
| Yes | 1261 (25.8%) | 247 (26.8%) | 150 (26.7%) | 150 (30.8%) | 81 (22.9%) |  |  |  |
| Alcohol, n (%) |  |  |  |  |  | 0.011 | 5.3% | 1.4% |
| No | 3638 (74.3%) | 691 (75.1%) | 431 (76.7%) | 352 (72.3%) | 293 (83.0%) |  |  |  |
| Yes | 1183 (24.2%) | 209 (22.7%) | 128 (22.8%) | 131 (26.9%) | 60 (17.0%) |  |  |  |
| Comorbidities, n (%) |  |  |  |  |  |  |  |  |
| Coronary Heart Disease |  |  |  |  |  | <0.001 | 6.7% | 0.0% |
| No | 3912 (79.9%) | 834 (90.7%) | 513 (91.3%) | 461 (94.7%) | 298 (84.4%) |  |  |  |
| Yes | 982 (20.1%) | 86 (9.3%) | 49 (8.7%) | 26 (5.3%) | 55 (15.6%) |  |  |  |
| Hypertension |  |  |  |  |  | <0.001 | 5.4% | 0.0% |
| No | 2231 (45.6%) | 557 (60.5%) | 301 (53.6%) | 281 (57.7%) | 163 (46.2%) |  |  |  |
| Yes | 2663 (54.4%) | 363 (39.5%) | 261 (46.4%) | 206 (42.3%) | 190 (53.8%) |  |  |  |
| Diabetes |  |  |  |  |  | <0.001 | 8.4% | 0.0% |
| No | 3645 (74.5%) | 817 (88.8%) | 489 (87.0%) | 412 (84.6%) | 263 (74.5%) |  |  |  |
| Yes | 1249 (25.5%) | 103 (11.2%) | 73 (13.0%) | 75 (15.4%) | 90 (25.5%) |  |  |  |
| Anemia |  |  |  |  |  | <0.001 | 1.3% | 0.0% |
| No | 4724 (96.5%) | 913 (99.2%) | 556 (98.9%) | 486 (99.8%) | 350 (99.2%) |  |  |  |
| Yes | 170 (3.5%) | 7 (0.8%) | 6 (1.1%) | 1 (0.2%) | 3 (0.8%) |  |  |  |
| Liver Dysfunction |  |  |  |  |  | <0.001 | 2.3% | 0.0% |
| No | 4613 (94.3%) | 913 (99.2%) | 548 (97.5%) | 485 (99.6%) | 351 (99.4%) |  |  |  |
| Yes | 281 (5.7%) | 7 (0.8%) | 14 (2.5%) | 2 (0.4%) | 2 (0.6%) |  |  |  |
| Renal Dysfunction |  |  |  |  |  | <0.001 | 2.4% | 0.0% |
| No | 4699 (96.0%) | 863 (93.8%) | 543 (96.6%) | 484 (99.4%) | 331 (93.8%) |  |  |  |
| Yes | 195 (4.0%) | 57 (6.2%) | 19 (3.4%) | 3 (0.6%) | 22 (6.2%) |  |  |  |
| Malignant Tumor |  |  |  |  |  | <0.001 | 22.1% | 0.0% |
| No | 2353 (48.1%) | 891 (96.8%) | 517 (92.0%) | 466 (95.7%) | 307 (87.0%) |  |  |  |
| Yes | 2541 (51.9%) | 29 (3.2%) | 45 (8.0%) | 21 (4.3%) | 46 (13.0%) |  |  |  |
| MET Score, n (%) |  |  |  |  |  | <0.001 | 27.5% | 15.8% |
| 1 | 90 (1.8%) | 44 (4.8%) | 2 (0.4%) | 7 (1.4%) | 18 (5.1%) |  |  |  |
| 2 | 175 (3.6%) | 48 (5.2%) | 12 (2.1%) | 19 (3.9%) | 19 (5.4%) |  |  |  |
| 3 | 124 (2.5%) | 61 (6.6%) | 10 (1.8%) | 7 (1.4%) | 32 (9.1%) |  |  |  |
| 4 | 541 (11.1%) | 136 (14.8%) | 21 (3.7%) | 57 (11.7%) | 29 (8.2%) |  |  |  |
| 5 | 1211 (24.7%) | 116 (12.6%) | 55 (9.8%) | 36 (7.4%) | 63 (17.8%) |  |  |  |
| 6 | 906 (18.5%) | 288 (31.3%) | 195 (34.7%) | 84 (17.2%) | 59 (16.7%) |  |  |  |
| 7 | 322 (6.6%) | 81 (8.8%) | 74 (13.2%) | 42 (8.6%) | 40 (11.3%) |  |  |  |
| 8 | 318 (6.5%) | 111 (12.1%) | 159 (28.3%) | 185 (38.0%) | 35 (9.9%) |  |  |  |
| 9 | 120 (2.5%) | 4 (0.4%) | 15 (2.7%) | 40 (8.2%) | 10 (2.8%) |  |  |  |
| 10 | 42 (0.9%) | 2 (0.2%) | 2 (0.4%) | 2 (0.4%) | 4 (1.1%) |  |  |  |
| Laboratory Tests (Median (IQR)) |  |  |  |  |  |  |  |  |
| WBC count, ×10⁹/L | 5.79 (4.83, 6.99) | 5.83 (4.87, 7.04) | 5.36 (4.37, 6.61) | 6.76 (5.00, 11.00) | 6.47 (5.38, 7.82) | <0.001 | 9.3% | 3.5% |
| Hemoglobin, g/L | 130.00 (119.00, 141.00) | 128.00 (115.00, 138.00) | 121.00 (109.00, 132.25) | 132.00 (116.25, 145.00) | 132.00 (121.00, 145.00) | <0.001 | 3.5% | 2.4% |
| ALT, U/L | 14.30 (10.50, 20.40) | 15.50 (11.00, 23.83) | 17.00 (12.00, 25.00) | 16.20 (11.55, 22.15) | 16.60 (12.30, 24.70) | <0.001 | 6.7% | 3.1% |
| AST, U/L | 16.50 (13.70, 20.80) | 18.70 (15.50, 23.60) | 22.00 (17.00, 27.00) | 20.30 (16.85, 25.90) | 17.50 (14.00, 22.95) | <0.001 | 11.6% | 4.2% |
| Glucose, mmol/L | 5.31 (4.84, 6.11) | 5.08 (4.68, 5.68) | 5.05 (4.66, 5.69) | 4.93 (4.50, 5.68) | 5.42 (4.82, 6.71) | <0.001 | 3.9% | 9.7% |
| Serum albumin, g/L | 40.40 (38.00, 43.00) | 38.20 (35.40, 40.82) | 37.65 (35.10, 40.10) | 42.20 (38.30, 45.50) | 40.95 (37.00, 43.90) | <0.001 | 4.8% | 2.7% |
| Urea, mmol/L | 5.36 (4.47, 6.49) | 6.12 (4.88, 7.33) | 5.54 (4.51, 6.66) | 5.41 (4.30, 6.63) | 5.80 (4.80, 7.10) | <0.001 | 5.5% | 3.5% |
| Serum creatinine, μmol/L | 71.90 (61.60, 84.20) | 71.40 (60.00, 86.15) | 70.75 (59.88, 86.83) | 65.00 (53.00, 80.00) | 62.70 (52.30, 77.15) | <0.001 | 6.2% | 2.7% |
| K, mmol/L | 3.97 (3.77, 4.21) | 4.06 (3.82, 4.28) | 4.00 (3.78, 4.25) | 4.08 (3.80, 4.36) | 4.13 (3.85, 4.41) | <0.001 | 1.6% | 3.0% |
| Na, mmol/L | 141.30 (139.70, 142.70) | 141.80 (140.20, 143.25) | 142.00 (140.40, 143.20) | 141.56 (140.00, 142.95) | 141.90 (139.90, 143.50) | <0.001 | 0.2% | 3.0% |
| Ca, mmol/L | 2.27 (2.20, 2.34) | 2.22 (2.14, 2.30) | 2.20 (2.13, 2.27) | 2.20 (2.11, 2.28) | 2.38 (2.29, 2.45) | <0.001 | 3.4% | 5.9% |
| Analgesic Use Within 24 Hours Preoperative, n (%) |  |  |  |  |  | <0.001 | 2.1% | 0.0% |
| No | 4656 (95.1%) | 913 (99.2%) | 562 (100.0%) | 487 (100.0%) | 352 (99.7%) |  |  |  |
| Yes | 238 (4.9%) | 7 (0.8%) | 0 (0.0%) | 0 (0.0%) | 1 (0.3%) |  |  |  |
| Surgical Department, n (%) |  |  |  |  |  | <0.001 | 6.0% | 0.0% |
| Head, neck, otorhinolaryngology and surgery | 229 (4.7%) | 111 (12.1%) | 14 (2.5%) | 29 (6.0%) | 5 (1.4%) |  |  |  |
| General surgery | 1750 (35.8%) | 318 (34.6%) | 200 (35.6%) | 178 (36.6%) | 105 (29.7%) |  |  |  |
| Thoracic Surgery | 629 (12.9%) | 115 (12.5%) | 73 (13.0%) | 34 (7.0%) | 29 (8.2%) |  |  |  |
| Urology | 582 (11.9%) | 207 (22.5%) | 156 (27.8%) | 118 (24.2%) | 67 (19.0%) |  |  |  |
| Gynecology | 169 (3.5%) | 36 (3.9%) | 23 (4.1%) | 0 (0.0%) | 3 (0.8%) |  |  |  |
| Orthopedics | 1404 (28.7%) | 132 (14.3%) | 90 (16.0%) | 117 (24.0%) | 142 (40.2%) |  |  |  |
| Vascular surgery | 131 (2.7%) | 1 (0.1%) | 6 (1.1%) | 11 (2.3%) | 2 (0.6%) |  |  |  |
| Type of Surgery, n (%) |  |  |  |  |  | <0.001 | 15.7% | 4.5% |
| Open Surgery | 2401 (49.1%) | 352 (38.3%) | 191 (34.0%) | 173 (35.5%) | 208 (58.9%) |  |  |  |
| Laparoscopic Surgery | 1951 (39.9%) | 452 (49.1%) | 272 (48.4%) | 182 (37.4%) | 141 (39.9%) |  |  |  |
| Other Surgery | 263 (5.4%) | 91 (9.9%) | 93 (16.5%) | 119 (24.4%) | 3 (0.8%) |  |  |  |
| Surgical Site, n (%) |  |  |  |  |  | <0.001 | 11.1% | 6.1% |
| Head and Neck | 385 (7.9%) | 125 (13.6%) | 25 (4.4%) | 22 (4.5%) | 22 (6.2%) |  |  |  |
| Upper Limbs | 109 (2.2%) | 5 (0.5%) | 8 (1.4%) | 14 (2.9%) | 15 (4.2%) |  |  |  |
| Trunk | 3069 (62.7%) | 656 (71.3%) | 373 (66.4%) | 268 (55.0%) | 259 (73.4%) |  |  |  |
| Lower limbs, buttocks and others | 912 (18.6%) | 123 (13.4%) | 151 (26.9%) | 178 (36.6%) | 57 (16.1%) |  |  |  |
| Malignant Tumor Surgery, n (%) |  |  |  |  |  | <0.001 | 21.3% | 2.1% |
| No | 2604 (53.2%) | 635 (69.0%) | 305 (54.3%) | 412 (84.6%) | 197 (55.8%) |  |  |  |
| Yes | 2266 (46.3%) | 262 (28.5%) | 166 (29.5%) | 68 (14.0%) | 148 (41.9%) |  |  |  |
| Surgery Grade, n (%) |  |  |  |  |  | <0.001 | 23.4% | 7.1% |
| I | 242 (4.9%) | 35 (3.8%) | 11 (2.0%) | 50 (10.3%) | 2 (0.6%) |  |  |  |
| II | 1711 (35.0%) | 326 (35.4%) | 103 (18.3%) | 345 (70.8%) | 54 (15.3%) |  |  |  |
| III | 1826 (37.3%) | 465 (50.5%) | 145 (25.8%) | 86 (17.7%) | 70 (19.8%) |  |  |  |
| IV | 644 (13.2%) | 69 (7.5%) | 295 (52.5%) | 2 (0.4%) | 224 (63.5%) |  |  |  |
| Surgical Incision Classification, n (%) |  |  |  |  |  | <0.001 | 13.0% | 7.2% |
| Natural Orifice | 82 (1.7%) | 35 (3.8%) | 20 (3.6%) | 83 (17.0%) | 31 (8.8%) |  |  |  |
| I | 1724 (35.2%) | 94 (10.2%) | 147 (26.2%) | 271 (55.6%) | 167 (47.3%) |  |  |  |
| II | 2439 (49.8%) | 457 (49.7%) | 363 (64.6%) | 112 (23.0%) | 150 (42.5%) |  |  |  |
| III | 159 (3.2%) | 291 (31.6%) | 21 (3.7%) | 7 (1.4%) | 1 (0.3%) |  |  |  |
| IV | 24 (0.5%) | 13 (1.4%) | 4 (0.7%) | 0 (0.0%) | 2 (0.6%) |  |  |  |
| Number of Surgical Incisions, n (%) |  |  |  |  |  | <0.001 | 23.8% | 11.2% |
| 1 | 2019 (41.3%) | 557 (60.5%) | 232 (41.3%) | 178 (36.6%) | 211 (59.8%) |  |  |  |
| 2 | 418 (8.5%) | 132 (14.3%) | 25 (4.4%) | 36 (7.4%) | 27 (7.6%) |  |  |  |
| 3 | 701 (14.3%) | 68 (7.4%) | 84 (14.9%) | 161 (33.1%) | 17 (4.8%) |  |  |  |
| 4 | 433 (8.8%) | 18 (2.0%) | 71 (12.6%) | 10 (2.1%) | 39 (11.0%) |  |  |  |
| 5 | 499 (10.2%) | 21 (2.3%) | 53 (9.4%) | 1 (0.2%) | 39 (11.0%) |  |  |  |
| 6 and above | 298 (6.1%) | 23 (2.5%) | 18 (3.2%) | 2 (0.4%) | 16 (4.5%) |  |  |  |
| Number of Drainage Tubes, n (%) |  |  |  |  |  | <0.001 | 19.3% | 12.5% |
| 0 | 969 (19.8%) | 217 (23.6%) | 104 (18.5%) | 206 (42.3%) | 74 (21.0%) |  |  |  |
| 1 | 2530 (51.7%) | 497 (54.0%) | 286 (50.9%) | 194 (39.8%) | 250 (70.8%) |  |  |  |
| 2 | 561 (11.5%) | 112 (12.2%) | 65 (11.6%) | 18 (3.7%) | 21 (5.9%) |  |  |  |
| 3 and above | 136 (2.8%) | 24 (2.6%) | 39 (6.9%) | 3 (0.6%) | 5 (1.4%) |  |  |  |
| Blood Loss, ml (Median (IQR)) | 50.00 (30.00, 200.00) | 50.00 (20.00, 200.00) | 100.00 (50.00, 200.00) | 20.00 (10.00, 100.00) | 66.50 (20.00, 200.00) | <0.001 | 50.9% | 10.3% |
| Surgery Duration, mins (Median (IQR)) | 138.50 (95.00, 205.00) | 140.00 (90.00, 215.00) | 130.00 (75.75, 206.25) | 110.00 (65.00, 190.00) | 150.00 (90.00, 230.00) | <0.001 | 11.2% | 0.6% |
| Urine Volume, ml (Median (IQR)) | 300.00 (100.00, 600.00) | 400.00 (200.00, 800.00) | 500.00 (400.00, 800.00) | 400.00 (300.00, 700.00) | 500.00 (300.00, 800.00) | <0.001 | 19.9% | 16.7% |
| Crystalloid, ml (Median (IQR)) | 1600.00 (1100.00, 2100.00) | 1000.00 (600.00, 1137.50) | 1000.00 (700.00, 1500.00) | 1100.00 (750.00, 1600.00) | 1200.00 (900.00, 1700.00) | <0.001 | 21.1% | 3.3% |
| ASA Classification, n (%) |  |  |  |  |  | <0.001 | 17.3% | 2.2% |
| I | 60 (1.2%) | 13 (1.4%) | 6 (1.1%) | 50 (10.3%) | 2 (0.6%) |  |  |  |
| II | 3854 (78.7%) | 441 (47.9%) | 356 (63.3%) | 338 (69.4%) | 231 (65.4%) |  |  |  |
| III | 937 (19.1%) | 379 (41.2%) | 139 (24.7%) | 93 (19.1%) | 111 (31.4%) |  |  |  |
| IV | 19 (0.4%) | 17 (1.8%) | 2 (0.4%) | 1 (0.2%) | 8 (2.3%) |  |  |  |
| Anesthesia Method, n (%) |  |  |  |  |  | <0.001 | 10.2% | 0.0% |
| Basic Anesthesia | 13 (0.3%) | 13 (1.4%) | 7 (1.2%) | 17 (3.5%) | 0 (0.0%) |  |  |  |
| Regional Nerve Block | 130 (2.7%) | 5 (0.5%) | 2 (0.4%) | 5 (1.0%) | 14 (4.0%) |  |  |  |
| Intraspinal Anesthesia | 86 (1.8%) | 6 (0.7%) | 11 (2.0%) | 3 (0.6%) | 63 (17.8%) |  |  |  |
| General Anesthesia | 4531 (92.6%) | 881 (95.8%) | 530 (94.3%) | 416 (85.4%) | 261 (73.9%) |  |  |  |
| Combined General Anesthesia | 134 (2.7%) | 15 (1.6%) | 12 (2.1%) | 46 (9.4%) | 15 (4.2%) |  |  |  |
| Intraoperative medication, n (%) |  |  |  |  |  |  |  |  |
| NSAIDs |  |  |  |  |  | <0.001 | 31.6% | 0.0% |
| No | 2458 (50.2%) | 848 (92.2%) | 562 (100.0%) | 234 (48.0%) | 336 (95.2%) |  |  |  |
| Yes | 2436 (49.8%) | 72 (7.8%) | 0 (0.0%) | 253 (52.0%) | 17 (4.8%) |  |  |  |
| Benzodiazepines |  |  |  |  |  | <0.001 | 20.2% | 0.0% |
| No | 1822 (37.2%) | 213 (23.2%) | 562 (100.0%) | 307 (63.0%) | 260 (73.7%) |  |  |  |
| Yes | 3072 (62.8%) | 707 (76.8%) | 0 (0.0%) | 180 (37.0%) | 93 (26.3%) |  |  |  |
| Ketamine |  |  |  |  |  | <0.001 | 3.9% | 0.0% |
| No | 4894 (100.0%) | 841 (91.4%) | 562 (100.0%) | 487 (100.0%) | 352 (99.7%) |  |  |  |
| Yes | 0 (0.0%) | 79 (8.6%) | 0 (0.0%) | 0 (0.0%) | 1 (0.3%) |  |  |  |
| Opioid Analgesics |  |  |  |  |  | <0.001 | 7.3% | 0.0% |
| No | 119 (2.4%) | 51 (5.5%) | 560 (99.6%) | 21 (4.3%) | 63 (17.8%) |  |  |  |
| Yes | 4775 (97.6%) | 869 (94.5%) | 2 (0.4%) | 466 (95.7%) | 290 (82.2%) |  |  |  |
| Local Anesthetics |  |  |  |  |  | <0.001 | 26.0% | 0.0% |
| No | 2565 (52.4%) | 393 (42.7%) | 562 (100.0%) | 367 (75.4%) | 263 (74.5%) |  |  |  |
| Yes | 2329 (47.6%) | 527 (57.3%) | 0 (0.0%) | 120 (24.6%) | 90 (25.5%) |  |  |  |
| Ondansetron |  |  |  |  |  | <0.001 | 24.1% | 0.0% |
| No | 2580 (52.7%) | 771 (83.8%) | 562 (100.0%) | 487 (100.0%) | 262 (74.2%) |  |  |  |
| Yes | 2314 (47.3%) | 149 (16.2%) | 0 (0.0%) | 0 (0.0%) | 91 (25.8%) |  |  |  |
| Tropisetron |  |  |  |  |  | <0.001 | 19.1% | 0.0% |
| No | 4026 (82.3%) | 918 (99.8%) | 562 (100.0%) | 300 (61.6%) | 353 (100.0%) |  |  |  |
| Yes | 868 (17.7%) | 2 (0.2%) | 0 (0.0%) | 187 (38.4%) | 0 (0.0%) |  |  |  |
| Dexamethasone |  |  |  |  |  | <0.001 | 7.1% | 0.0% |
| No | 4827 (98.6%) | 912 (99.1%) | 562 (100.0%) | 408 (83.8%) | 346 (98.0%) |  |  |  |
| Yes | 67 (1.4%) | 8 (0.9%) | 0 (0.0%) | 79 (16.2%) | 7 (2.0%) |  |  |  |
| Methylprednisolone |  |  |  |  |  | <0.001 | 23.1% | 0.0% |
| No | 2578 (52.7%) | 916 (99.6%) | 562 (100.0%) | 487 (100.0%) | 326 (92.4%) |  |  |  |
| Yes | 2316 (47.3%) | 4 (0.4%) | 0 (0.0%) | 0 (0.0%) | 27 (7.6%) |  |  |  |
| Flurbiprofen |  |  |  |  |  | <0.001 | 26.2% | 0.0% |
| No | 3205 (65.5%) | 849 (92.3%) | 562 (100.0%) | 236 (48.5%) | 337 (95.5%) |  |  |  |
| Yes | 1689 (34.5%) | 71 (7.7%) | 0 (0.0%) | 251 (51.5%) | 16 (4.5%) |  |  |  |
| Parecoxib |  |  |  |  |  | <0.001 | 5.0% | 0.0% |
| No | 4350 (88.9%) | 919 (99.9%) | 562 (100.0%) | 485 (99.6%) | 352 (99.7%) |  |  |  |
| Yes | 544 (11.1%) | 1 (0.1%) | 0 (0.0%) | 2 (0.4%) | 1 (0.3%) |  |  |  |
| Ketorolac |  |  |  |  |  | <0.001 | 2.3% | 0.0% |
| No | 4644 (94.9%) | 920 (100.0%) | 562 (100.0%) | 487 (100.0%) | 353 (100.0%) |  |  |  |
| Yes | 250 (5.1%) | 0 (0.0%) | 0 (0.0%) | 0 (0.0%) | 0 (0.0%) |  |  |  |
| Opioid Antagonists |  |  |  |  |  | <0.001 | 17.6% | 0.0% |
| No | 4853 (99.2%) | 582 (63.3%) | 562 (100.0%) | 487 (100.0%) | 350 (99.2%) |  |  |  |
| Yes | 41 (0.8%) | 338 (36.7%) | 0 (0.0%) | 0 (0.0%) | 3 (0.8%) |  |  |  |
| Postoperative Analgesia, n (%) |  |  |  |  |  | <0.001 | 22.2% | 0.0% |
| No | 2048 (41.8%) | 509 (55.3%) | 464 (82.6%) | 275 (56.5%) | 301 (85.3%) |  |  |  |
| Yes | 2846 (58.2%) | 411 (44.7%) | 98 (17.4%) | 212 (43.5%) | 52 (14.7%) |  |  |  |
| POD, n (%) |  |  |  |  |  | <0.001 | 5.5% | 0.0% |
| No | 4149 (84.8%) | 862 (93.7%) | 470 (83.6%) | 437 (89.7%) | 333 (94.3%) |  |  |  |
| Yes | 745 (15.2%) | 58 (6.3%) | 92 (16.4%) | 50 (10.3%) | 20 (5.7%) |  |  |  |
| AKI, n (%) |  |  |  |  |  | 0.144 | 1.1% | 0.0% |
| No | 4614 (94.3%) | 876 (95.2%) | 541 (96.3%) | 467 (95.9%) | 331 (93.8%) |  |  |  |
| Yes | 280 (5.7%) | 44 (4.8%) | 21 (3.7%) | 20 (4.1%) | 22 (6.2%) |  |  |  |

Continuous variables are presented as median (IQR) and were compared across centers using the Kruskal-Wallis test. Categorical variables are presented as n (%) and were compared using the chi-square test. P values indicate between-center comparisons across the five participating centers. Missing (%) indicates the overall missing rate for each variable across all five centers, and CV (%) indicates the coefficient of variation across centers.

**Table S2. Participant characteristics when building POD model**

| **Variable** | **Training Dataset (N=5489)** | **Internal Validation Dataset (N=1374)** | **P_1_** | **External Validation Dataset (N=353)** | **P_2_** |
| --- | --- | --- | --- | --- | --- |
| Hospital |  |  |  |  |  |
| Chinese PLA General Hospital | 3915 (71.3) | 979 (71.3) |  |  |  |
| The Second Xiangya Hospital of Central South University | 736 (13.4) | 184 (13.4) |  |  |  |
| Union Hospital, Tongji Medical College, Huazhong University of Science and Technology | 449 (8.2) | 113 (8.2) |  |  |  |
| The Affiliated Hospital of Guizhou Medical University | 389 (7.1) | 98 (7.1) |  |  |  |
| Beijing Tiantan Hospital, Capital Medical University |  |  |  | 353 (100) |  |
| Sex, n (%) |  |  | 0.085 |  | 0.094 |
| female | 2508 (45.7) | 664 (48.3) |  | 178 (50.4) |  |
| male | 2981 (54.3) | 710 (51.7) |  | 175 (49.6) |  |
| Age, years (Median (IQR)) | 71 (68,75) | 71 (68,75) | 0.922 | 71 (67,75) | 0.915 |
| BMI, kg/m^2^ (Median (IQR)) | 24.2 (22.2,26.2) | 24.2 (22.3,26.4) | 0.497 | 24.5 (22.6,27) | 0.031 |
| Educational Level, n (%) |  |  | 0.594 |  | <0.001 |
| Below primary school | 480 (8.7) | 137 (10) |  | 22 (6.2) |  |
| Primary school | 1347 (24.5) | 321 (23.4) |  | 62 (17.6) |  |
| Junior high school | 1471 (26.8) | 358 (26.1) |  | 123 (34.8) |  |
| High school (including technical secondary school) | 1014 (18.5) | 257 (18.7) |  | 80 (22.7) |  |
| College, junior college | 1149 (20.9) | 291 (21.2) |  | 62 (17.6) |  |
| Postgraduate or above | 28 (0.5) | 10 (0.7) |  | 4 (1.1) |  |
| Smoke, n (%) | 1489 (27.1) | 326 (23.7) | 0.012 | 81 (22.9) | 0.098 |
| Alcohol, n (%) | 1369 (24.9) | 304 (22.1) | 0.032 | 60 (17) | 0.001 |
| Comorbidities, n (%) |  |  |  |  |  |
| Coronary Heart Disease | 903 (16.5) | 240 (17.5) | 0.388 | 55 (15.6) | 0.723 |
| Hypertension | 2792 (50.9) | 701 (51) | 0.943 | 190 (53.8) | 0.306 |
| Diabetes | 1214 (22.1) | 286 (20.8) | 0.314 | 90 (25.5) | 0.158 |
| Anemia | 157 (2.9) | 27 (2) | 0.081 | 3 (0.8) | 0.018 |
| Liver Dysfunction | 233 (4.2) | 71 (5.2) | 0.158 | 2 (0.6) | <0.001 |
| Renal Dysfunction | 226 (4.1) | 48 (3.5) | 0.327 | 22 (6.2) | 0.076 |
| Malignant Tumor | 2109 (38.4) | 527 (38.4) | 0.988 | 46 (13) | <0.001 |
| MET Score, n (%) |  |  | 0.138 |  | <0.001 |
| 1 | 104 (1.9) | 39 (2.8) |  | 18 (5.1) |  |
| 2 | 209 (3.8) | 57 (4.1) |  | 19 (5.4) |  |
| 3 | 195 (3.6) | 50 (3.6) |  | 32 (9.1) |  |
| 4 | 834 (15.2) | 184 (13.4) |  | 39 (11) |  |
| 5 | 1572 (28.6) | 381 (27.7) |  | 88 (24.9) |  |
| 6 | 1363 (24.8) | 351 (25.5) |  | 68 (19.3) |  |
| 7 | 421 (7.7) | 103 (7.5) |  | 40 (11.3) |  |
| 8 | 608 (11.1) | 165 (12) |  | 35 (9.9) |  |
| 9 | 139 (2.5) | 40 (2.9) |  | 10 (2.8) |  |
| 10 | 44 (0.8) | 4 (0.3) |  | 4 (1.1) |  |
| Laboratory Tests (Median (IQR)) |  |  |  |  |  |
| WBC count, *10^9^·L^-1^ | 5.83 (4.83,7.08) | 5.8 (4.88,6.975) | 0.801 | 6.47 (5.4,7.81) | <0.001 |
| Hemoglobin, g·L^-1^ | 129 (118,140) | 130 (118,140) | 0.798 | 132 (121,145) | 0.003 |
| ALT, u·L^-1^ | 14.9 (10.8,21.4) | 14.75 (10.9,21) | 0.587 | 16.7 (12.5,24.7) | <0.001 |
| AST, u·L^-1^ | 17.5 (14.3,22.3) | 17.612 (14.3,22.2) | 0.592 | 18.01 (14,22.9) | 0.745 |
| Glucose, mmol·L^-1^ | 5.31 (4.83,6.03) | 5.29 (4.83,5.99) | 0.734 | 5.46 (4.9,6.66) | 0.005 |
| Serum albumin, g·L^-1^ | 40.1 (37.4,42.8) | 40.1 (37.6,42.6) | 0.631 | 41 (37,43.7) | 0.069 |
| Urea, mmol·L^-1^ | 5.46 (4.52,6.57) | 5.54 (4.62,6.70) | 0.054 | 5.7 (4.9,7.1) | <0.001 |
| Serum creatinine, μmol·L^-1^ | 71 (61.2,84) | 70.9 (61.2,83.8) | 0.798 | 63.4 (52.7,77.4) | <0.001 |
| K, mmol·L^-1^ | 3.99 (3.79,4.23) | 3.99 (3.78,4.22) | 0.398 | 4.13 (3.86,4.4) | <0.001 |
| Na, mmol·L^-1^ | 141.4 (139.9,142.8) | 141.4 (139.9,142.7) | 0.857 | 141.8 (140,143.5) | 0.101 |
| Ca, mmol·L^-1^ | 2.26 (2.19,2.33) | 2.26 (2.19,2.33) | 0.489 | 2.36 (2.25,2.44) | <0.001 |
| Analgesic Use Within 24 Hours Preoperative, n (%) | 186 (3.4) | 59 (4.3) | 0.124 | 1 (0.3) | <0.001 |
| Surgical Department, n (%) |  |  | 0.776 |  | <0.001 |
| Head, neck, otorhinolaryngology and surgery | 310 (5.6) | 73 (5.3) |  | 5 (1.4) |  |
| General surgery | 1950 (35.5) | 496 (36.1) |  | 105 (29.7) |  |
| Thoracic Surgery | 697 (12.7) | 154 (11.2) |  | 29 (8.2) |  |
| Urology | 852 (15.5) | 211 (15.4) |  | 67 (19) |  |
| Gynecology | 179 (3.3) | 49 (3.6) |  | 3 (0.8) |  |
| Orthopedics | 1381 (25.2) | 362 (26.3) |  | 142 (40.2) |  |
| Vascular surgery | 120 (2.2) | 29 (2.1) |  | 2 (0.6) |  |
| Type of Surgery, n (%) |  |  | 0.468 |  | <0.001 |
| Open Surgery | 2668 (48.6) | 676 (49.2) |  | 208 (58.9) |  |
| Laparoscopic Surgery | 2356 (42.9) | 570 (41.5) |  | 142 (40.2) |  |
| Other Surgery | 465 (8.5) | 128 (9.3) |  | 3 (0.8) |  |
| Surgical Site, n (%) |  |  | 0.12 |  | 0.002 |
| Head and Neck | 502 (9.1) | 115 (8.4) |  | 22 (6.2) |  |
| Upper Limbs | 113 (2.1) | 29 (2.1) |  | 15 (4.2) |  |
| Trunk | 3746 (68.2) | 908 (66.1) |  | 259 (73.4) |  |
| Lower limbs, buttocks and others | 1128 (20.6) | 322 (23.4) |  | 57 (16.1) |  |
| Malignant Tumor Surgery, n (%) | 2242 (40.8) | 558 (40.6) | 0.899 | 148 (41.9) | 0.73 |
| Surgery Grade, n (%) |  |  | 0.023 |  | <0.001 |
| Ⅰ | 338 (6.2) | 63 (4.6) |  | 3 (0.8) |  |
| Ⅱ | 2318 (42.2) | 585 (42.6) |  | 54 (15.3) |  |
| Ⅲ | 2052 (37.4) | 497 (36.2) |  | 72 (20.4) |  |
| Ⅳ | 781 (14.2) | 229 (16.7) |  | 224 (63.5) |  |
| Surgical Incision Classification, n (%) |  |  | 0.989 |  | <0.001 |
| Natural Orifice | 229 (4.2) | 56 (4.1) |  | 31 (8.8) |  |
| Ⅰ | 2114 (38.5) | 531 (38.6) |  | 168 (47.6) |  |
| Ⅱ | 2735 (49.8) | 679 (49.4) |  | 151 (42.8) |  |
| Ⅲ | 378 (6.9) | 100 (7.3) |  | 1 (0.3) |  |
| Ⅳ | 33 (0.6) | 8 (0.6) |  | 2 (0.6) |  |
| Number of Surgical Incisions, n (%) |  |  | 0.359 |  | <0.001 |
| 1 | 2522 (45.9) | 641 (46.7) |  | 213 (60.3) |  |
| 2 | 761 (13.9) | 193 (14) |  | 29 (8.2) |  |
| 3 | 970 (17.7) | 219 (15.9) |  | 17 (4.8) |  |
| 4 | 472 (8.6) | 116 (8.4) |  | 39 (11) |  |
| 5 | 504 (9.2) | 123 (9) |  | 39 (11) |  |
| 6 and above | 260 (4.7) | 82 (6) |  | 16 (4.5) |  |
| Number of Drainage Tubes, n (%) |  |  | 0.053 |  | <0.001 |
| 0 | 1683 (30.7) | 458 (33.3) |  | 77 (21.8) |  |
| 1 | 3020 (55) | 739 (53.8) |  | 250 (70.8) |  |
| 2 | 612 (11.1) | 149 (10.8) |  | 21 (5.9) |  |
| 3 and above | 174 (3.2) | 28 (2) |  | 5 (1.4) |  |
| Blood Loss, ml (Median (IQR)) | 69.0 (30,200) | 69.8 (26.3,200) | 0.915 | 80 (20,200) | 0.864 |
| Surgery Duration, mins (Median (IQR)) | 135 (90,205) | 135 (90,202.25) | 0.608 | 150 (90,231) | 0.093 |
| Urine Volume, ml (Median (IQR)) | 300 (100,600) | 300 (100,600) | 0.512 | 500 (200,800) | <0.001 |
| Crystalloid, ml (Median (IQR)) | 1300 (1000,1800) | 1300 (1000,1900) | 0.586 | 1200 (900,1700) | <0.001 |
| ASA Classification, n (%) |  |  | 0.22 |  | <0.001 |
| Ⅰ | 108 (2) | 28 (2) |  | 2 (0.6) |  |
| Ⅱ | 4139 (75.4) | 999 (72.7) |  | 232 (65.7) |  |
| Ⅲ | 1212 (22.1) | 338 (24.6) |  | 111 (31.4) |  |
| Ⅳ | 30 (0.5) | 9 (0.7) |  | 8 (2.3) |  |
| Anesthesia Method, n (%) |  |  | 0.249 |  | <0.001 |
| Basic Anesthesia | 39 (0.7) | 11 (0.8) |  | 0 |  |
| Regional Nerve Block | 112 (2) | 30 (2.2) |  | 14 (4) |  |
| Intraspinal Anesthesia | 89 (1.6) | 17 (1.2) |  | 63 (17.8) |  |
| General Anesthesia | 5072 (92.4) | 1286 (93.6) |  | 261 (73.9) |  |
| Combined General Anesthesia | 177 (3.2) | 30 (2.2) |  | 15 (4.2) |  |
| Intraoperative medication, n (%) |  |  |  |  |  |
| NSAIDs | 2173 (39.6) | 588 (42.8) | 0.033 | 17 (4.8) | <0.001 |
| Benzodiazepines | 3165 (57.7) | 794 (57.8) | 0.957 | 93 (26.3) | <0.001 |
| Ketamine | 69 (1.3) | 10 (0.7) | 0.133 | 1 (0.3) | 0.128 |
| Opioid Analgesics | 4893 (89.1) | 1219 (88.7) | 0.689 | 290 (82.2) | <0.001 |
| Local Anesthetics | 2392 (43.6) | 584 (42.5) | 0.491 | 90 (25.5) | <0.001 |
| Ondansetron | 1957 (35.7) | 506 (36.8) | 0.436 | 91 (25.8) | <0.001 |
| Tropisetron | 832 (15.2) | 225 (16.4) | 0.282 | 0 | <0.001 |
| Dexamethasone | 123 (2.2) | 31 (2.3) | >0.999 | 7 (2) | 0.895 |
| Methylprednisolone | 1836 (33.4) | 484 (35.2) | 0.225 | 27 (7.6) | <0.001 |
| Flurbiprofen | 1581 (28.8) | 430 (31.3) | 0.075 | 16 (4.5) | <0.001 |
| Parecoxib | 437 (8) | 110 (8) | >0.999 | 1 (0.3) | <0.001 |
| Ketorolac | 194 (3.5) | 56 (4.1) | 0.38 | 0 | <0.001 |
| Opioid Antagonists | 299 (5.4) | 80 (5.8) | 0.632 | 3 (0.8) | <0.001 |
| Postoperative Analgesia, n (%) | 2861 (52.1) | 706 (51.4) | 0.645 | 52 (14.7) | <0.001 |
| POD, n (%) | 756 (13.8) | 189 (13.8) | >0.999 | 20 (5.7) | <0.001 |

P1, training dataset vs. internal validation dataset; P2, training dataset vs. external validation dataset.

**Table S3. Participant characteristics when building AKI model**

| **Variable** | **Training Dataset (N=5489)** | **Internal Validation Dataset (N=1374)** | **P_1_** | **External Validation Dataset (N=353)** | **P_2_** |
| --- | --- | --- | --- | --- | --- |
| Hospital |  |  |  |  |  |
| Chinese PLA General Hospital | 3915 (71.3) | 979 (71.3) |  |  |  |
| The Second Xiangya Hospital of Central South University | 736 (13.4) | 184 (13.4) |  |  |  |
| Union Hospital, Tongji Medical College, Huazhong University of Science and Technology | 449 (8.2) | 113 (8.2) |  |  |  |
| The Affiliated Hospital of Guizhou Medical University | 389 (7.1) | 98 (7.1) |  |  |  |
| Beijing Tiantan Hospital, Capital Medical University |  |  |  | 353 (100) |  |
| Sex, n (%) |  |  | 0.696 |  | 0.127 |
| female | 2530 (46.1) | 642 (46.7) |  | 178 (50.4) |  |
| male | 2959 (53.9) | 732 (53.3) |  | 175 (49.6) |  |
| Age, years (Median (IQR)) | 71 (68,75) | 71 (68,74) | 0.856 | 71 (67,75) | 0.928 |
| BMI, kg/m^2^ (Median (IQR)) | 24.2 (22.3,26.2) | 24.2 (22.2,26.2) | 0.566 | 24.329 (22.6,27) | 0.078 |
| Educational Level, n (%) |  |  | 0.946 |  | 0.001 |
| Below primary school | 484 (8.8) | 118 (8.6) |  | 22 (6.2) |  |
| Primary school | 1312 (23.9) | 342 (24.9) |  | 62 (17.6) |  |
| Junior high school | 1477 (26.9) | 360 (26.2) |  | 123 (34.8) |  |
| High school (including technical secondary school) | 1023 (18.6) | 249 (18.1) |  | 79 (22.4) |  |
| College, junior college | 1160 (21.1) | 298 (21.7) |  | 63 (17.8) |  |
| Postgraduate or above | 33 (0.6) | 7 (0.5) |  | 4 (1.1) |  |
| Smoke, n (%) | 1451 (26.4) | 362 (26.3) | 0.974 | 81 (22.9) | 0.167 |
| Alcohol, n (%) | 1331 (24.2) | 344 (25) | 0.567 | 60 (17) | 0.002 |
| Comorbidities, n (%) |  |  |  |  |  |
| Coronary Heart Disease | 897 (16.3) | 246 (17.9) | 0.177 | 55 (15.6) | 0.763 |
| Hypertension | 2808 (51.2) | 685 (49.9) | 0.405 | 190 (53.8) | 0.359 |
| Diabetes | 1215 (22.1) | 285 (20.7) | 0.28 | 90 (25.5) | 0.16 |
| Anemia | 141 (2.6) | 43 (3.1) | 0.29 | 3 (0.8) | 0.048 |
| Liver Dysfunction | 227 (4.1) | 77 (5.6) | 0.022 | 2 (0.6) | <0.001 |
| Renal Dysfunction | 217 (4) | 57 (4.1) | 0.8 | 22 (6.2) | 0.05 |
| Malignant Tumor | 2108 (38.4) | 528 (38.4) | >0.999 | 46 (13) | <0.001 |
| MET Score, n (%) |  |  | 0.351 |  | <0.001 |
| 1 | 112 (2) | 31 (2.3) |  | 18 (5.1) |  |
| 2 | 201 (3.7) | 55 (4) |  | 19 (5.4) |  |
| 3 | 184 (3.4) | 48 (3.5) |  | 32 (9.1) |  |
| 4 | 743 (13.5) | 197 (14.3) |  | 35 (9.9) |  |
| 5 | 1717 (31.3) | 436 (31.7) |  | 89 (25.2) |  |
| 6 | 1295 (23.6) | 323 (23.5) |  | 70 (19.8) |  |
| 7 | 418 (7.6) | 103 (7.5) |  | 41 (11.6) |  |
| 8 | 643 (11.7) | 130 (9.5) |  | 35 (9.9) |  |
| 9 | 143 (2.6) | 36 (2.6) |  | 10 (2.8) |  |
| 10 | 33 (0.6) | 15 (1.1) |  | 4 (1.1) |  |
| Laboratory Tests (Median (IQR)) |  |  |  |  |  |
| WBC count, *10^9^·L^-1^ | 5.85 (4.83,7.06) | 5.84 (4.9,7.078) | 0.559 | 6.46 (5.4,7.79) | <0.001 |
| Hemoglobin, g·L^-1^ | 129.684 (118,140) | 130 (118,140) | 0.66 | 132 (121,145) | 0.003 |
| ALT, u·L^-1^ | 14.8 (10.8,21.2) | 14.985 (10.9,21.1) | 0.765 | 16.7 (12.5,24.6) | <0.001 |
| AST, u·L^-1^ | 17.4 (14.3,22) | 17.666 (14.4,22.3) | 0.595 | 17.7 (14,22.7) | 0.645 |
| Glucose, mmol·L^-1^ | 5.32 (4.84,5.99) | 5.313 (4.8,5.96) | 0.568 | 5.46 (4.9,6.66) | 0.004 |
| Serum albumin, g·L^-1^ | 40 (37.4,42.7) | 40.3 (37.7,42.7) | 0.132 | 40.7 (37,43.7) | 0.083 |
| Urea, mmol·L^-1^ | 5.446 (4.52,6.57) | 5.511 (4.622,6.688) | 0.021 | 5.632 (4.991,7) | <0.001 |
| Serum creatinine, μmol·L^-1^ | 71.2 (61.2,83.9) | 71.65 (61.5,84.322) | 0.401 | 63.4 (52.7,76.8) | <0.001 |
| K, mmol·L^-1^ | 3.993 (3.78,4.23) | 3.997 (3.782,4.23) | 0.937 | 4.13 (3.86,4.4) | <0.001 |
| Na, mmol·L^-1^ | 141.4 (139.9,142.8) | 141.4 (139.9,142.8) | 0.727 | 141.8 (140,143.5) | 0.08 |
| Ca, mmol·L^-1^ | 2.259 (2.19,2.32) | 2.26 (2.19,2.32) | 0.717 | 2.35 (2.26,2.44) | <0.001 |
| Analgesic Use Within 24 Hours Preoperative, n (%) | 199 (3.6) | 46 (3.3) | 0.678 | 1 (0.3) | <0.001 |
| Surgical Department, n (%) |  |  | 0.268 |  | <0.001 |
| Head, neck, otorhinolaryngology and surgery | 314 (5.7) | 69 (5) |  | 5 (1.4) |  |
| General surgery | 1984 (36.1) | 462 (33.6) |  | 105 (29.7) |  |
| Thoracic Surgery | 667 (12.2) | 184 (13.4) |  | 29 (8.2) |  |
| Urology | 854 (15.6) | 209 (15.2) |  | 67 (19) |  |
| Gynecology | 181 (3.3) | 47 (3.4) |  | 3 (0.8) |  |
| Orthopedics | 1377 (25.1) | 366 (26.6) |  | 142 (40.2) |  |
| Vascular surgery | 112 (2) | 37 (2.7) |  | 2 (0.6) |  |
| Type of Surgery, n (%) |  |  | 0.677 |  | <0.001 |
| Open Surgery | 2686 (48.9) | 656 (47.7) |  | 208 (58.9) |  |
| Laparoscopic Surgery | 2340 (42.6) | 595 (43.3) |  | 142 (40.2) |  |
| Other Surgery | 463 (8.4) | 123 (9) |  | 3 (0.8) |  |
| Surgical Site, n (%) |  |  | 0.206 |  | 0.002 |
| Head and Neck | 490 (8.9) | 106 (7.7) |  | 22 (6.2) |  |
| Upper Limbs | 116 (2.1) | 21 (1.5) |  | 15 (4.2) |  |
| Trunk | 3728 (67.9) | 941 (68.5) |  | 259 (73.4) |  |
| Lower limbs, buttocks and others | 1155 (21) | 306 (22.3) |  | 57 (16.1) |  |
| Malignant Tumor Surgery, n (%) | 2232 (40.7) | 562 (40.9) | 0.896 | 149 (42.2) | 0.605 |
| Surgery Grade, n (%) |  |  | 0.667 |  | <0.001 |
| Ⅰ | 338 (6.2) | 73 (5.3) |  | 2 (0.6) |  |
| Ⅱ | 2305 (42) | 575 (41.8) |  | 55 (15.6) |  |
| Ⅲ | 2039 (37.1) | 523 (38.1) |  | 72 (20.4) |  |
| Ⅳ | 807 (14.7) | 203 (14.8) |  | 224 (63.5) |  |
| Surgical Incision Classification, n (%) |  |  | 0.699 |  | <0.001 |
| Natural Orifice | 219 (4) | 54 (3.9) |  | 31 (8.8) |  |
| Ⅰ | 2141 (39) | 552 (40.2) |  | 169 (47.9) |  |
| Ⅱ | 2719 (49.5) | 659 (48) |  | 150 (42.5) |  |
| Ⅲ | 380 (6.9) | 98 (7.1) |  | 1 (0.3) |  |
| Ⅳ | 30 (0.5) | 11 (0.8) |  | 2 (0.6) |  |
| Number of Surgical Incisions, n (%) |  |  | 0.337 |  | <0.001 |
| 1 | 2588 (47.1) | 625 (45.5) |  | 211 (59.8) |  |
| 2 | 738 (13.4) | 192 (14) |  | 29 (8.2) |  |
| 3 | 911 (16.6) | 246 (17.9) |  | 17 (4.8) |  |
| 4 | 480 (8.7) | 137 (10) |  | 39 (11) |  |
| 5 | 497 (9.1) | 108 (7.9) |  | 41 (11.6) |  |
| 6 and above | 275 (5) | 66 (4.8) |  | 16 (4.5) |  |
| Number of Drainage Tubes, n (%) |  |  | 0.291 |  | <0.001 |
| 0 | 1692 (30.8) | 412 (30) |  | 77 (21.8) |  |
| 1 | 3010 (54.8) | 787 (57.3) |  | 250 (70.8) |  |
| 2 | 619 (11.3) | 141 (10.3) |  | 21 (5.9) |  |
| 3 and above | 168 (3.1) | 34 (2.5) |  | 5 (1.4) |  |
| Blood Loss, ml (Median (IQR)) | 60 (27.45,200) | 60 (30,200) | 0.958 | 80 (20,200) | 0.935 |
| Surgery Duration, mins (Median (IQR)) | 135 (90,205) | 137.5 (94.25,200) | 0.73 | 150 (90,231) | 0.082 |
| Urine Volume, ml (Median (IQR)) | 300 (100,600) | 305.007 (100,600) | 0.149 | 500 (200,800) | <0.001 |
| Crystalloid, ml (Median (IQR)) | 1300 (1000,1900) | 1300 (1000,1800) | 0.68 | 1200 (900,1700) | <0.001 |
| ASA Classification, n (%) |  |  | 0.244 |  | <0.001 |
| Ⅰ | 104 (1.9) | 26 (1.9) |  | 2 (0.6) |  |
| Ⅱ | 4102 (74.7) | 1043 (75.9) |  | 232 (65.7) |  |
| Ⅲ | 1247 (22.7) | 302 (22) |  | 111 (31.4) |  |
| Ⅳ | 36 (0.7) | 3 (0.2) |  | 8 (2.3) |  |
| Anesthesia Method, n (%) |  |  | 0.092 |  | <0.001 |
| Basic Anesthesia | 33 (0.6) | 17 (1.2) |  | 0 |  |
| Regional Nerve Block | 108 (2) | 34 (2.5) |  | 14 (4) |  |
| Intraspinal Anesthesia | 83 (1.5) | 23 (1.7) |  | 63 (17.8) |  |
| General Anesthesia | 5101 (92.9) | 1257 (91.5) |  | 261 (73.9) |  |
| Combined General Anesthesia | 164 (3) | 43 (3.1) |  | 15 (4.2) |  |
| Intraoperative medication, n (%) |  |  |  |  |  |
| NSAIDs | 2235 (40.7) | 526 (38.3) | 0.106 | 17 (4.8) | <0.001 |
| Benzodiazepines | 3186 (58) | 773 (56.3) | 0.243 | 93 (26.3) | <0.001 |
| Ketamine | 61 (1.1) | 18 (1.3) | 0.634 | 1 (0.3) | 0.182 |
| Opioid Analgesics | 4894 (89.2) | 1218 (88.6) | 0.619 | 290 (82.2) | <0.001 |
| Local Anesthetics | 2355 (42.9) | 621 (45.2) | 0.133 | 90 (25.5) | <0.001 |
| Ondansetron | 1978 (36) | 485 (35.3) | 0.633 | 91 (25.8) | <0.001 |
| Tropisetron | 858 (15.6) | 199 (14.5) | 0.311 | 0 | <0.001 |
| Dexamethasone | 116 (2.1) | 38 (2.8) | 0.174 | 7 (2) | >0.999 |
| Methylprednisolone | 1852 (33.7) | 468 (34.1) | 0.847 | 27 (7.6) | <0.001 |
| Flurbiprofen | 1642 (29.9) | 369 (26.9) | 0.028 | 16 (4.5) | <0.001 |
| Parecoxib | 431 (7.9) | 116 (8.4) | 0.505 | 1 (0.3) | <0.001 |
| Ketorolac | 198 (3.6) | 52 (3.8) | 0.816 | 0 | <0.001 |
| Opioid Antagonists | 302 (5.5) | 77 (5.6) | 0.934 | 3 (0.8) | <0.001 |
| Postoperative Analgesia, n (%) | 2837 (51.7) | 730 (53.1) | 0.353 | 52 (14.7) | <0.001 |
| AKI, n (%) | 292 (5.3) | 73 (5.3) | >0.999 | 22 (6.2) | 0.538 |

P1, training dataset vs. internal validation dataset; P2, training dataset vs. external validation dataset.

**Table S4. Performance of POD Federated Learning Models in 10-fold Cross-validation**.

| **Model** |  | **Acc** | **Sensitivity** | **Specificity** | **AUC** | **AUC 95%CI** |
| --- | --- | --- | --- | --- | --- | --- |
| FedAvg | 1 | 0.683 | 0.484 | 0.715 | 0.662 | 0.602-0.718 |
|  | 2 | 0.642 | 0.632 | 0.644 | 0.696 | 0.640-0.748 |
|  | 3 | 0.645 | 0.568 | 0.657 | 0.681 | 0.622-0.738 |
|  | 4 | 0.699 | 0.589 | 0.716 | 0.702 | 0.652-0.753 |
|  | 5 | 0.644 | 0.526 | 0.663 | 0.646 | 0.580-0.705 |
|  | 6 | 0.698 | 0.521 | 0.726 | 0.661 | 0.595-0.724 |
|  | 7 | 0.636 | 0.511 | 0.655 | 0.635 | 0.577-0.694 |
|  | 8 | 0.695 | 0.574 | 0.714 | 0.700 | 0.644-0.752 |
|  | 9 | 0.645 | 0.670 | 0.641 | 0.692 | 0.633-0.748 |
|  | 10 | 0.653 | 0.617 | 0.658 | 0.710 | 0.656-0.765 |
| FedLSD | 1 | 0.686 | 0.505 | 0.715 | 0.662 | 0.603-0.718 |
|  | 2 | 0.647 | 0.632 | 0.649 | 0.697 | 0.640-0.750 |
|  | 3 | 0.639 | 0.568 | 0.650 | 0.681 | 0.620-0.737 |
|  | 4 | 0.700 | 0.589 | 0.718 | 0.702 | 0.652-0.753 |
|  | 5 | 0.646 | 0.526 | 0.665 | 0.645 | 0.579-0.704 |
|  | 6 | 0.700 | 0.521 | 0.728 | 0.662 | 0.595-0.724 |
|  | 7 | 0.637 | 0.500 | 0.659 | 0.634 | 0.576-0.693 |
|  | 8 | 0.695 | 0.564 | 0.716 | 0.699 | 0.642-0.750 |
|  | 9 | 0.650 | 0.702 | 0.641 | 0.692 | 0.633-0.748 |
|  | 10 | 0.653 | 0.617 | 0.658 | 0.710 | 0.655-0.764 |
| FedProx | 1 | 0.686 | 0.495 | 0.717 | 0.662 | 0.602-0.718 |
|  | 2 | 0.641 | 0.632 | 0.642 | 0.696 | 0.641-0.748 |
|  | 3 | 0.645 | 0.568 | 0.657 | 0.681 | 0.622-0.738 |
|  | 4 | 0.699 | 0.589 | 0.716 | 0.702 | 0.652-0.753 |
|  | 5 | 0.644 | 0.526 | 0.663 | 0.646 | 0.580-0.705 |
|  | 6 | 0.697 | 0.521 | 0.725 | 0.661 | 0.595-0.724 |
|  | 7 | 0.636 | 0.511 | 0.655 | 0.635 | 0.577-0.694 |
|  | 8 | 0.693 | 0.574 | 0.712 | 0.701 | 0.644-0.752 |
|  | 9 | 0.645 | 0.670 | 0.641 | 0.692 | 0.632-0.748 |
|  | 10 | 0.653 | 0.617 | 0.658 | 0.710 | 0.656-0.765 |

**Table S5. Calibration performance of centralized and federated learning models for POD.**

| **Dataset** | **Model** | **Calibration intercept** | **Calibration slope** | **Brier score** |
| --- | --- | --- | --- | --- |
| Internal validation | Centralized | -0.009 | 0.932 | 0.108 |
|  | FedAvg | 0.024 | 1.043 | 0.109 |
|  | FedLSD | 0.024 | 1.064 | 0.109 |
|  | FedProx | 0.023 | 1.045 | 0.109 |
| External validation | Centralized | -1.231 | 0.940 | 0.068 |
|  | FedAvg | -1.125 | 0.904 | 0.064 |
|  | FedLSD | -1.119 | 0.941 | 0.064 |
|  | FedProx | -1.126 | 0.909 | 0.064 |

Calibration metrics were calculated using the beta-calibrated probabilities used to generate the corresponding calibration curves. An intercept of 0 and a slope of 1 indicate ideal calibration; lower Brier scores indicate lower overall probabilistic prediction error.

**Table S6. Validation performance of sensitivity analysis models for POD prediction.**

| **Dataset** | **Model** | **Acc** | **Sensitivity** | **Specificity** | **AUC** | **AUC 95%CI** |
| --- | --- | --- | --- | --- | --- | --- |
| Internal validation | Central learning (full variables) | 0.726 | 0.608 | 0.745 | 0.732 | 0.693-0.769 |
|  | Age+ASA | 0.664 | 0.455 | 0.697 | 0.612 | 0.566-0.655 |
|  | Preoperative variables | 0.680 | 0.529 | 0.705 | 0.683 | 0.639-0.724 |
| External validation | Central learning (full variables) | 0.629 | 0.650 | 0.628 | 0.706 | 0.599-0.817 |
|  | Age+ASA | 0.691 | 0.550 | 0.700 | 0.568 | 0.402-0.712 |
|  | Preoperative variables | 0.601 | 0.450 | 0.610 | 0.584 | 0.466-0.697 |

The Age + ASA model included only age and ASA classification. The preoperative-only model included presurgical variables only, including demographics, comorbidities, BMI, ASA classification, preoperative MET score, and surgical department/site; intraoperative and postoperative variables were excluded.

**Table S7. Performance of AKI Federated Learning Models in 10-fold Cross-validation.**

| **Model** |  | **Acc** | **Sensitivity** | **Specificity** | **AUC** | **AUC 95%CI** |
| --- | --- | --- | --- | --- | --- | --- |
| FedAvg | 1 | 0.708 | 0.778 | 0.704 | 0.780 | 0.710-0.834 |
|  | 2 | 0.711 | 0.649 | 0.714 | 0.753 | 0.677-0.824 |
|  | 3 | 0.718 | 0.722 | 0.717 | 0.812 | 0.752-0.873 |
|  | 4 | 0.691 | 0.778 | 0.687 | 0.755 | 0.672-0.832 |
|  | 5 | 0.714 | 0.639 | 0.718 | 0.768 | 0.690-0.839 |
|  | 6 | 0.672 | 0.694 | 0.671 | 0.701 | 0.612-0.779 |
|  | 7 | 0.681 | 0.784 | 0.675 | 0.817 | 0.748-0.879 |
|  | 8 | 0.664 | 0.676 | 0.664 | 0.743 | 0.664-0.820 |
|  | 9 | 0.679 | 0.649 | 0.681 | 0.726 | 0.639-0.808 |
|  | 10 | 0.654 | 0.811 | 0.645 | 0.783 | 0.715-0.844 |
| FedLSD | 1 | 0.708 | 0.778 | 0.704 | 0.780 | 0.710-0.836 |
|  | 2 | 0.709 | 0.649 | 0.713 | 0.755 | 0.678-0.825 |
|  | 3 | 0.719 | 0.722 | 0.719 | 0.813 | 0.753-0.874 |
|  | 4 | 0.693 | 0.778 | 0.688 | 0.755 | 0.672-0.832 |
|  | 5 | 0.713 | 0.639 | 0.717 | 0.768 | 0.690-0.839 |
|  | 6 | 0.671 | 0.694 | 0.669 | 0.701 | 0.610-0.779 |
|  | 7 | 0.679 | 0.784 | 0.673 | 0.816 | 0.747-0.878 |
|  | 8 | 0.664 | 0.730 | 0.660 | 0.746 | 0.666-0.822 |
|  | 9 | 0.676 | 0.649 | 0.677 | 0.725 | 0.638-0.808 |
|  | 10 | 0.654 | 0.811 | 0.645 | 0.783 | 0.714-0.843 |
| FedProx | 1 | 0.708 | 0.778 | 0.704 | 0.779 | 0.709-0.834 |
|  | 2 | 0.711 | 0.649 | 0.714 | 0.753 | 0.677-0.824 |
|  | 3 | 0.718 | 0.722 | 0.717 | 0.813 | 0.751-0.874 |
|  | 4 | 0.693 | 0.778 | 0.688 | 0.755 | 0.671-0.832 |
|  | 5 | 0.714 | 0.639 | 0.718 | 0.768 | 0.690-0.839 |
|  | 6 | 0.672 | 0.694 | 0.671 | 0.701 | 0.611-0.779 |
|  | 7 | 0.682 | 0.784 | 0.676 | 0.817 | 0.747-0.878 |
|  | 8 | 0.667 | 0.676 | 0.667 | 0.743 | 0.663-0.819 |
|  | 9 | 0.679 | 0.649 | 0.681 | 0.725 | 0.639-0.808 |
|  | 10 | 0.654 | 0.811 | 0.645 | 0.783 | 0.715-0.844 |

**Table S8. Calibration performance of centralized and federated learning models for AKI.**

| **Dataset** | **Model** | **Calibration intercept** | **Calibration slope** | **Brier score** |
| --- | --- | --- | --- | --- |
| Internal validation | Centralized | 0.004 | 0.726 | 0.047 |
|  | FedAvg | -0.025 | 0.363 | 0.048 |
|  | FedLSD | -0.025 | 0.368 | 0.048 |
|  | FedProx | -0.025 | 0.362 | 0.048 |
| External validation | Centralized | -0.067 | 0.436 | 0.064 |
|  | FedAvg | 0.076 | 0.255 | 0.062 |
|  | FedLSD | 0.064 | 0.251 | 0.063 |
|  | FedProx | 0.076 | 0.255 | 0.062 |

Calibration metrics were calculated using the beta-calibrated probabilities used to generate the corresponding calibration curves. An intercept of 0 and a slope of 1 indicate ideal calibration; lower Brier scores indicate lower overall probabilistic prediction error.

**Table S9. Validation performance of sensitivity analysis models for AKI prediction.**

| **Dataset** | **Model** | **Acc** | **Sensitivity** | **Specificity** | **AUC** | **AUC 95%CI** |
| --- | --- | --- | --- | --- | --- | --- |
| Internal validation | Central learning (full variables) | 0.641 | 0.849 | 0.630 | 0.786 | 0.727-0.837 |
|  | Age+ASA | 0.715 | 0.562 | 0.724 | 0.667 | 0.600-0.730 |
|  | Preoperative variables | 0.686 | 0.603 | 0.691 | 0.721 | 0.662-0.775 |
| External validation | Central learning (full variables) | 0.609 | 0.773 | 0.598 | 0.733 | 0.646-0.815 |
|  | Age+ASA | 0.623 | 0.500 | 0.631 | 0.560 | 0.415-0.696 |
|  | Preoperative variables | 0.700 | 0.455 | 0.716 | 0.586 | 0.448-0.710 |

The Age + ASA model included only age and ASA classification. The preoperative-only model included presurgical variables only, including demographics, comorbidities, BMI, ASA classification, preoperative MET score, and surgical department/site; intraoperative and postoperative variables were excluded.
